# Quantitative CRISPR interference screens in yeast identify chemical-genetic interactions and new rules for guide RNA design

**DOI:** 10.1186/s13059-016-0900-9

**Published:** 2016-03-08

**Authors:** Justin D. Smith, Sundari Suresh, Ulrich Schlecht, Manhong Wu, Omar Wagih, Gary Peltz, Ronald W. Davis, Lars M. Steinmetz, Leopold Parts, Robert P. St.Onge

**Affiliations:** Stanford Genome Technology Center, Department of Biochemistry, Stanford University, 3165 Porter Drive, Palo Alto, CA 94304 USA; Department of Genetics, Stanford University School of Medicine, Stanford, California USA; Department of Anesthesia, Stanford University School of Medicine, Stanford University, Stanford, California 94305 USA; European Molecular Biology Laboratory, European Bioinformatics Institute (EMBL-EBI), Genome Campus, Hinxton, CB101SD UK; European Molecular Biology Laboratory (EMBL), Genome Biology Unit, 69117 Heidelberg, Germany; Current address: Wellcome Trust Sanger Institute, Hinxton, CB101SA UK

**Keywords:** CRISPRi, dCas9, Yeast, *Saccharomyces cerevisiae*, Nucleosome, Chemical-genetic interactions

## Abstract

**Background:**

Genome-scale CRISPR interference (CRISPRi) has been used in human cell lines; however, the features of effective guide RNAs (gRNAs) in different organisms have not been well characterized. Here, we define rules that determine gRNA effectiveness for transcriptional repression in *Saccharomyces cerevisiae*.

**Results:**

We create an inducible single plasmid CRISPRi system for gene repression in yeast, and use it to analyze fitness effects of gRNAs under 18 small molecule treatments. Our approach correctly identifies previously described chemical-genetic interactions, as well as a new mechanism of suppressing fluconazole toxicity by repression of the ERG25 gene. Assessment of multiple target loci across treatments using gRNA libraries allows us to determine generalizable features associated with gRNA efficacy. Guides that target regions with low nucleosome occupancy and high chromatin accessibility are clearly more effective. We also find that the best region to target gRNAs is between the transcription start site (TSS) and 200 bp upstream of the TSS. Finally, unlike nuclease-proficient Cas9 in human cells, the specificity of truncated gRNAs (18 nt of complementarity to the target) is not clearly superior to full-length gRNAs (20 nt of complementarity), as truncated gRNAs are generally less potent against both mismatched and perfectly matched targets.

**Conclusions:**

Our results establish a powerful functional and chemical genomics screening method and provide guidelines for designing effective gRNAs, which consider chromatin state and position relative to the target gene TSS. These findings will enable effective library design and genome-wide programmable gene repression in many genetic backgrounds.

**Electronic supplementary material:**

The online version of this article (doi:10.1186/s13059-016-0900-9) contains supplementary material, which is available to authorized users.

## Background

The bacterial type II CRISPR (clustered regularly interspaced palindromic repeats) associated Cas9 nuclease can be targeted to DNA using an engineered guide RNA (gRNA), enabling genome editing in a variety of organisms [1–4]. The Cas9 protein can be further modified to act as a programmable effector. Two point mutations can yield a catalytically dead Cas9 (dCas9) [3] which alone can serve as an effective programmable transcriptional repressor in bacteria [5]. With further modification, dCas9 can be made to function as a transcription activator or repressor (aka CRISPR interference, or CRISPRi) capable of modulating gene expression in eukaryotes [6–10], including in *Saccharomyces cerevisiae* [6, 7]. One of the advantages of CRISPR/Cas9 over previous methods of genome engineering such as Transcription Activator Like Effector Nucleases (TALENs) and Zinc Fingers is the compatibility of the specificity-determining region of the gRNA (generally 20 bases in length) with highly-parallel array-based oligonucleotide synthesis. Thus, large libraries of gRNAs can be readily synthesized and cloned for functional genomic or genome editing applications. Several groups have taken advantage of this, and generated genome-wide libraries for knocking out [11–14], silencing [15], and activating genes [15, 16].

The tremendous potential of the CRISPR/Cas9 system has motivated efforts to better understand factors that influence its efficacy. Gilbert *et al.* [15] characterized the ideal genomic region to target gRNAs for effective repression in K562 human myeloid leukemia cells. They found CRISPRi worked best using gRNAs that direct dCas9-KRAB to a window of -50 bp to +300 bp relative to the transcription start site (TSS) of a gene, with a maximum effect observed in the 50-100 bp region just downstream of the TSS [15]. It is currently not known if these rules for guide positioning apply to other cell lines or organisms. Further, not all gRNAs targeted to this window functioned equally well, and therefore additional factors likely influence efficacy.

Understanding and limiting the off-target activity of CRISPR/Cas9 is also important for most applications of the system. Several groups have demonstrated that CRISPR/Cas9 can tolerate some mismatches between the gRNA and the target, indicating potential to cut or bind unintended sites [10, 17–21]. One strategy that has proven effective in preventing off-target cutting in human HEK293 and U2OS cells is to truncate the gRNA’s region of target site complementarity from 20 nt to 17 nt or 18 nt [20, 22]. The specificity of these truncated gRNAs has only been tested in human cells, however, and only with nuclease-proficient Cas9.

Here, we present a versatile platform for high-throughput characterization of CRISPR/Cas9 gRNA libraries in *Saccharomyces cerevisiae*. Informed by existing chemical-genomic data, we designed and tested gRNAs directed to 20 genes whose expression was predicted to influence sensitivity to specific small molecule inhibitors of growth. Repression of these genes by dCas9-Mxi1 indeed produced quantifiable and drug-specific growth defects, which we then used to assess a variety of factors potentially influencing efficacy and specificity. We evaluated the effect of genome position, chromatin accessibility, nucleosome and transcription factor occupancy of the target site, as well as the length, sequence, and secondary structure of the gRNA. While our major goal was to determine rules predictive of CRISPR/Cas9 function in yeast, our experiments also revealed surprising biological insights, including a novel cellular mechanism for resistance to the antifungal drug fluconazole. Collectively, our results advance the development of CRISPRi as a powerful approach for functional genomics.

## Results

### Single plasmid system for CRISPR interference in yeast

**Fig. 1:**
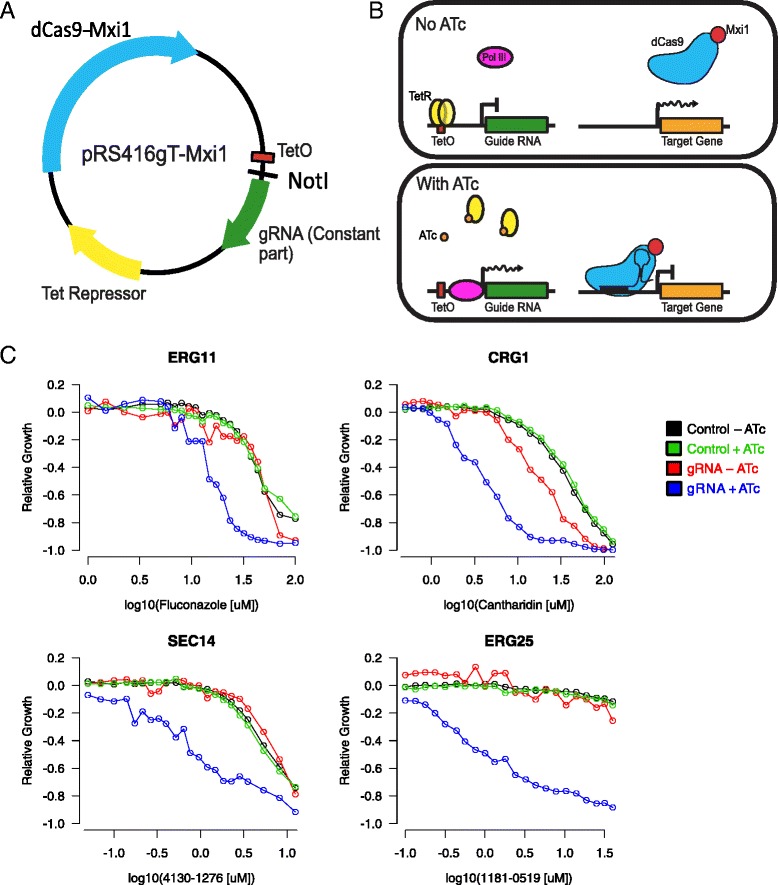
**a** Schematic of expression construct for regulatable CRISPRi in yeast. Key features include ORFs expressing dCas9-Mxi1 and the tetracycline repressor (TetR), as well as a tetracycline inducible gRNA locus containing the RPR1 promoter with a TetO site, a NotI site for cloning new gRNA sequences encoding target complementarity, and the constant part of the gRNA. **b** In the absence of anhydrotetracycline (ATc) TetR binds the gRNA promoter and prevents PolIII from binding and transcribing the gRNA. This in turn prevents dCas9-Mxi1 from binding the target site. In the presence of ATc, TetR dissociates and gRNA is expressed, allowing dCas9-Mxi1 to bind its target locus, and repress gene expression. **c** CRISPRi-induced drug-sensitivity. Transformants expressing gRNAs directed against *CRG1*, *ERG11*, *ERG25*, and *SEC14* (indicated above each panel), were grown in the presence of a specific small molecule (that is, cantharidin, fluconazole, 1181-0519, and 4130-1276, respectively). Growth of the gRNA-expressing strain and the empty vector control, was measured in the presence and absence of ATc (see legend). Growth relative to the ‘no-drug’ control is indicated on the y-axis (see Methods), in increasing concentrations of each small molecule (x-axis)

We designed and constructed a plasmid for regulatable CRISPRi in yeast (Fig. 1). The plasmid is a derivative of pRS416 [23], which contains a yeast centromeric origin of replication and a *URA3* selectable marker. To this backbone, we added the complete open reading frame (ORF) for catalytically inactive *Streptococcus pyogenes* Cas9 (dCas9) to which the Mxi1 transcriptional repressor was fused at the C-terminus [6]. We also added the tetracycline repressor (tetR) ORF, a tetO-modified RPR1 RNA polymerase III promoter [7, 24], a NotI restriction site, and common gRNA sequence. The NotI site enables rapid cloning of short oligonucleotides encoding the target complementarity region of the RNA guide. In this system, TetR and dCas9-Mxi1 are constitutively expressed from the *GPM1* and *TEF1* promoters, respectively, whereas the gRNA is inducibly expressed by addition of anhydrotetracycline (ATc) to the growth medium (Fig. 1b).

To validate this system as a rapid and versatile approach for transcriptional silencing in yeast, we designed gRNAs targeting the *ERG11*, *ERG25*, *CRG1*, and *SEC14* genes. Previous work has demonstrated that these four genes are haploinsufficient in the presence of the small molecule inhibitors fluconazole, 1181-0519, cantharidin, and 4130-1276 [25, 26], respectively. Thus, we reasoned that transcriptional repression by dCas9-Mxi1 should produce a growth defect in the presence of the appropriate chemical compound. Based on previous small scale studies [7], guides targeting regions near the TSS of each gene were synthesized, and inserted into the NotI site of our expression construct (Methods). The growth rates of transformants were then measured in increasing concentrations of the appropriate compounds, in both the presence and absence of ATc (Methods). In all four cases, and as expected, ATc-induced expression of the gRNA resulted in increased small molecule sensitivity relative to the empty-vector control (Fig. 1c, Additional file 1).

We characterized the system further and showed that ATc-dependent repression was titratable by addition of increasing concentrations of ATc to the culture (Additional file 2: Figure S1). Quantitative PCR (qPCR) analysis of transcript levels revealed rapid repression within approximately 2.5 h following ATc addition, but slow reversibility. Repression levels varied among the gRNAs assayed with the most effective gRNA repressing transcription roughly 10-fold. Even though we observed modest ATc-independent small molecule sensitivity for one of four gRNAs (*CRG1*) in Fig. 1c (possibly indicating leaky expression), qPCR analysis of *CRG1* transcript levels did not reveal significant gene repression in the non-induced (-ATc) condition (Additional file 2: Figure S1, Additional file 3). Thus, the collective data are consistent with strong transcriptional control of the guide.

### High-throughput CRISPRi via gRNA library screening

Akin to the DNA barcodes used for the yeast deletion collection [27, 28], the short specificity-determining regions of gRNAs (that is, the sequence complementary to the target) can act as unique identifiers of individual strains. Like barcodes, these can be readily quantified using next-generation sequencing [29, 30], thereby enabling high-throughput strain phenotyping following competitive growth in pooled cultures. By taking advantage of this, and inexpensive array-based oligonucleotide DNA synthesis, we sought to establish a quantitative assay for guide efficacy, with the goal of uncovering generalizable rules for effective use of CRISPRi in yeast (Additional file 2: Figure S2). In total, we created and tested five gRNA libraries comprised of a total of 989 unique gRNAs (Additional file 4), in the presence of various small molecule inhibitors of growth (Additional file 5). The guide counts following competitive growth were highly reproducible between biological replicates, indicating that the assay is robust (Additional file 2: Figure S3A-B, Additional files 6 and 7).

We first tested a library (that is, the ‘gene_tiling20’ library) of 238 guides targeting protospacer adjacent motif (PAM)-containing positions (on both the template and nontemplate strands) between 150 bp upstream of the TSS and +50 bp relative to the ORF start of 20 different genes. These 20 genes included the four described in Fig. 1c, and 16 others that have a specific small molecule partner that when added to a culture at the correct dose, will render that gene haploinsufficient [25]. Repression of a target gene is expected to increase sensitivity to its specific small molecule partner, but in general, not to other compounds under study. We leveraged this “reference set” of chemical genetic interactions (Additional file 8) to benchmark the assay and assess gRNA efficacy.

**Fig. 2:**
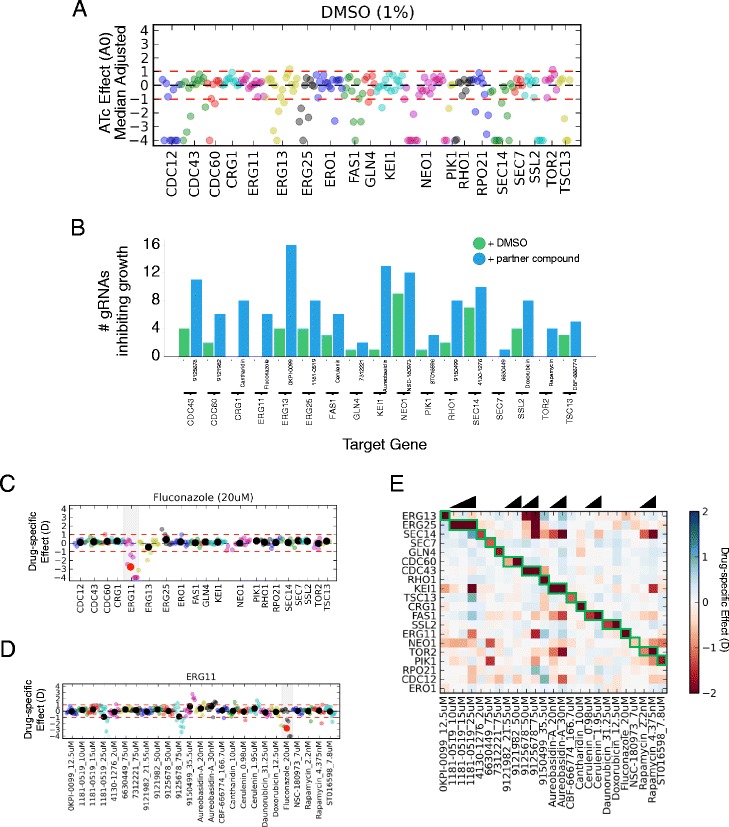
Parallel analysis of CRIPSRi-induced fitness defects in pooled cultures. **a** Effect of gRNA expression on growth. gRNA sequencing counts following growth in induced (+ATc) vs. uninduced (-ATc) conditions were used to calculate the ATc-effect (***A0***) for each gRNA, which were median-centered and plotted on the y-axis. Each point represents a gRNA directed against one of 20 different target genes (gene_tiling20 library). gRNAs are color-coded and arranged alphabetically on the x-axis by target gene. In the plot, ***A0*** values below -4 were set to -4. **b** Effect of small molecules on detecting gRNA-induced growth defects. For each gene target (x-axis), the number of gRNAs inducing a growth defect (median-centered ***A*** < -1) in standard conditions, and in the presence of its paired reference small molecule is plotted on the y-axis (see legend). **c** Fluconazole-specific growth defects (y-axis) are plotted for each gRNA (x-axis), which are color-coded and arranged alphabetically by target gene. The drug/gene pair representing the reference chemical-genetic interaction is highlighted in gray. **d** Drug-specific effects for the *ERG11* gRNA set in 25 different drug conditions (x-axis). Points are color-coded by condition. Large black dots represent the mean in each drug condition, and are colored red if >1 or if < -1. In **c** and **d**, drug-specific effect (***D***) values below -4 were set to -4. **e** Heatmap illustrating the average drug-specific effect for each guide set (y-axis), in each condition (x-axis). A guide set refers to the group of guides directed against the same gene. Drug-sensitivity is indicated in red, drug-resistance in blue. Previously defined chemical-genetic interactions are arranged on the diagonal and are outlined in green. Triangles above indicate cases where the same compound was assayed at increasing concentrations

All but one (*CRG1*) of the genes targeted by the ‘gene_tiling20’ library are essential for viability. Consistent with effective repression of an essential gene, several guides exhibited fewer sequencing reads following growth in the presence of ATc, compared to that in the absence of ATc (Fig. 2a). Notably, however, the majority of gRNAs targeting the 19 essential genes did not elicit a growth phenotype. Raw sequence read information, and the ATc-induced fold change (***A***; see Methods) of each gRNA, are listed in Additional files 9 and 10, respectively.

We challenged this library with 18 different small molecules from our reference set (Additional file 8), and consistently observed that addition of a small molecule to the culture increased the number of gRNAs causing growth defects. Specifically, in addition to those gRNAs that inhibit growth by virtue of their potent repression of an essential gene, additional gRNAs targeting genes known to be haploinsufficient for the added compound became depleted following competitive growth (Additional file 2: Figure S4A). For example, although no guides directed against the essential gene *ERG11* inhibited growth (median-adjusted ***A*** < -1) under standard conditions, six *ERG11* guides produced growth defects when yeast were cultured in 20 μM fluconazole, an antifungal drug that inhibits the Erg11 protein (Fig. 2b). These results were representative of the other compounds tested. In each case, the addition of compound increased assay sensitivity, allowing the effects of guides that only weakly modulate transcription to be detected (Fig. 2b). Nonetheless, not all guides produced a growth defect even when induced in the presence of their partner small molecule. Factors influencing guide efficacy are explored in detail later in the manuscript.

### Exploring small molecule mechanism-of-action

To specifically explore small molecule mechanism of action (MoA), we calculated the ‘drug-specific effect’ (***D***; see Methods) on each strain by comparing induced (+ATc) cultures grown in the presence of a small molecule, to those grown without the small molecule. This drug-induced fold change metric identifies only those genes that are dosage sensitive to the test compound. These genes are powerful descriptors of a compound’s MoA. For example, comparing relative guide counts following growth +/- 20 μM fluconazole primarily identifies strains in which *ERG11* is repressed as sensitive to fluconazole (Fig. 2c). Moreover, *ERG11*-repressor strains were, for the most part, not affected by the other compounds assayed (Fig. 2d). These results were representative of the other, previously-defined, chemical-genetic interactions that comprised our reference set (Additional file 11, Additional file 2: Figure S4B-C).

Collective analysis of strains expressing gRNAs directed against the same gene further verified that the small molecules tested specifically affected strains predicted by our reference set (Fig. 2e, Additional file 12). Interestingly, however, we also observed several examples where a small molecule affected the growth of a strain not predicted beforehand (off-diagonal red signal in Fig. 2e). As this figure reports the average drug-specific effect (***D***) on strains in a set, off-target binding by gRNAs is an unlikely explanation for the unexpected signal. Indeed, several lines of evidence suggest many represent *bona fide* chemical-genetic interactions. In cases where the same compound was tested at multiple concentrations, these interactions were reproducible and dose-dependent. The compound 9125678 was particularly interesting, as it inhibited growth of strains in which *ERG11*, *ERG13*, and *ERG25* were repressed. All three genes encode components of the yeast ergosterol pathway, suggesting a mechanism of action to be tested in future experiments.

**Fig. 3:**
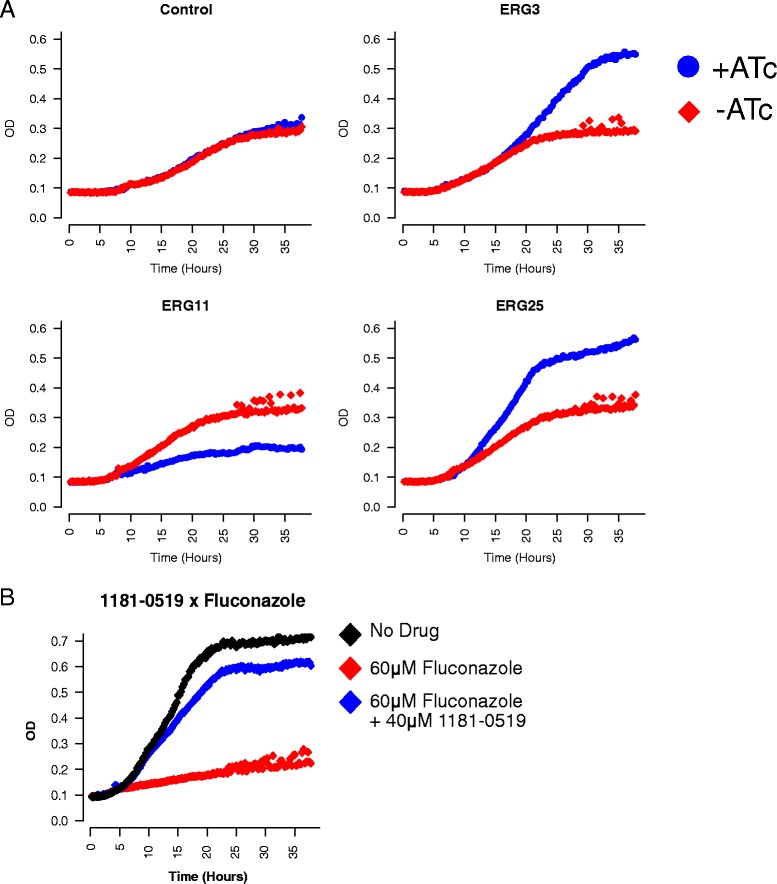
Erg25 regulates fluconazole sensitivity. **a** Strains containing gRNA constructs directed against *ERG3*, *ERG11*, *ERG25*, and the empty vector control (indicated above each panel), were grown in 63.1 μM fluconazole, in either the presence (blue), or absence of 250 ng/μL ATc (red). In each panel, optical density (OD) is plotted on the y-axis, as a function of time on the x-axis. Loss of *ERG3* function was previously shown to confer fluconazole resistance [55, 56] and served as a positive control. **b** Similar to (**a**). The parental BY4741 strain was grown in 60 μM fluconazole (red), 60 μM fluconazole + 40 μM 1181-0519 (blue), or no drug (black)

We also found that the growth-inhibitory effects of fluconazole were reduced in multiple *ERG25*-repressor strains, suggesting that *ERG25* repression confers resistance to fluconazole. Growth assays in isogenic cultures confirmed this observation (Fig. 3a, Additional file 13). Similar results were obtained via chemical inhibition of the Erg25 protein with 1181-0519. This compound, predicted by metabolomic profiling to inhibit Erg25 (Additional file 2: Figure S5 and Additional file 14), increased growth of the control strain (BY4741) in the presence of fluconazole (Fig. 3b). Interestingly, CRISPRi-mediated repression of *ERG25* caused an approximately 2.5-fold increase in *ERG11* transcript levels, thus providing a likely explanation for the observed fluconazole resistance (Additional file 2: Figure S1B and S1D, Additional file 3).

### Efficacy and specificity of full-length and truncated gRNAs

**Fig. 4:**
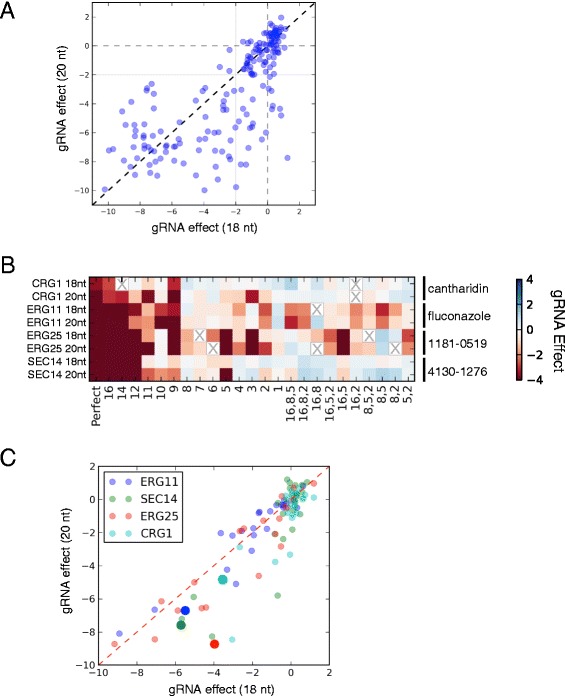
Quantitative comparison of full-length and truncated gRNAs. **a** gRNA effects (see methods) of 182 full-length gRNAs (20 nt of complementarity to the target) are plotted on the y-axis, and their truncated counterparts (18 nt of complementarity) on the x-axis. In all cases, gRNA-expressing strains were grown in the appropriate reference small molecule. Dotted and solid gray lines demarcate gRNA effects of 0 and -2, respectively. **b** Heatmaps illustrating growth defects induced by gRNAs containing different mismatches to the target sequence. Full-length and truncated gRNAs are arranged by target gene on the y-axis. The reference small molecule is labeled on the right. The mismatch position of each gRNA relative to the PAM is indicated on the x-axis (gRNAs matching the target sequence perfectly are on the far left). Missing values are indicated with an X. **c** As in (a), only the mismatched gRNAs described in (b) are plotted. Points are color-coded based on target gene (see legend). Large points represent the ‘perfect’ gRNAs, all other points represent gRNAs containing mismatches.

Having validated our overall approach and reference set, we next evaluated factors influencing gRNA performance. To this end, we focused on the growth inhibitory effects of gRNAs specifically in the presence of their partner chemical compound. Experiments in human cell lines have demonstrated that off-target cutting by Cas9 can be mitigated by reducing the length of the gRNA’s target complementarity from 20 nt, to either 17 or 18 nt [20, 22]. To assess effects of gRNA length on CRISPRi in yeast, we created an 18 nt version of our gene_tiling20 library described above (gene_tiling18), and assayed it under the same conditions. The growth effects resulting from both versions of each gRNA were generally consistent, with full-length and truncated versions of a gRNA often exhibiting similar effects (Fig. 4a). We found, however, that full-length gRNAs tended to produce stronger phenotypes more often: for example, 94 of 182 full-length but only 73 of 182 truncated gRNAs resulted in growth defects (gRNA effect < -2, solid gray lines in Fig. 4a).

To compare the specificity of gRNAs with 18 nt and 20 nt of target complementarity, we selected a single functional guide for *ERG11*, *ERG25*, *CRG1*, and *SEC14*, and designed a series of derivatives containing one, two, or three mismatches to the target sequence (24 in total for each target gene, for both truncated and full-length gRNAs). gRNA-induced sensitivity to the appropriate small molecule was assayed and, as expected, expression of the ‘perfect’ gRNA resulted in sensitivity (Fig. 4b, Additional file 2: Figure S6. As previously reported [17, 18, 21], we found that mismatches located in the seed region (that is, positions 1-10 relative to the PAM) were poorly tolerated by both full-length and truncated gRNAs (Fig. 4b, Additional file 2: Figure S6). In general, gRNAs containing mismatches in this region had reduced efficacy (that is, they did not yield growth defects), while mismatches in the distal region (positions 11-20) had little influence on efficacy. Plotting the effects of full-length and truncated gRNAs against each other (Fig. 4c) revealed that gRNAs with 20 nt of complementarity tended to be more effective repressors than those with 18 nt. Importantly however, this was true of both the perfect and mismatched gRNAs. Thus, considering their reduced efficacy against perfectly matching target sequences, truncated gRNAs did not exhibit a marked improvement in specificity compared to their full-length counterparts.

### gRNA efficacy depends on accessibility and location of the target region

**Fig. 5:**
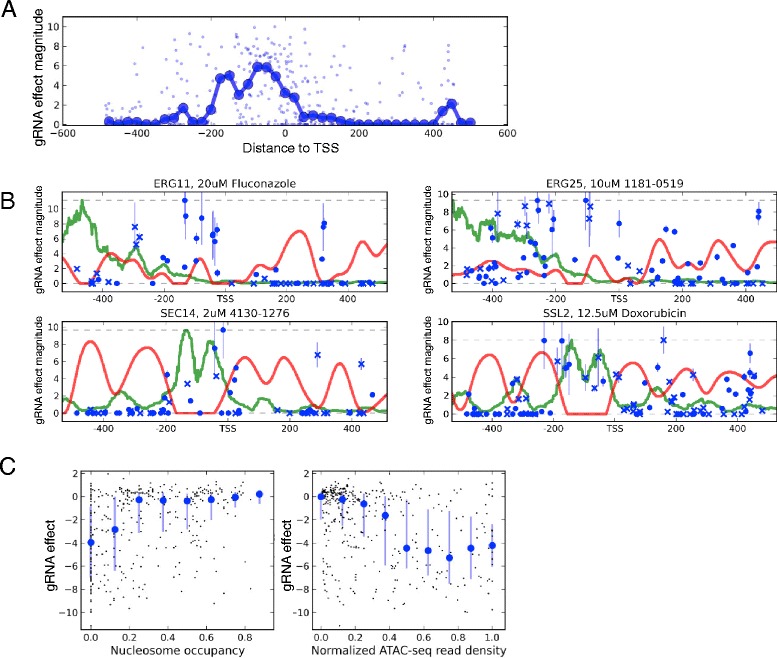
Effect of target location and accessibility on gRNA efficacy. In all plots gRNA efficacy was measured in the presence of the appropriate reference small molecule. **a** gRNA effect magnitude (absolute value of gRNA effects that were censored to have a maximum of 0) is plotted on the y-axis, against target position relative to the TSS on the x-axis. The median in 50 bp windows (solid line, big markers), overlapping by 25 bp, indicates a region of 200 bp immediately upstream the TSS as effective. **b** gRNA effect magnitude is plotted on the y-axis, against target position (gRNA midpoint) relative to the TSS on the x-axis for four loci (indicated above each plot). gRNAs targeting template and non-template strands are indicated with ‘o’ and ‘x’, respectively. Standard deviation estimates are indicated with blue lines, and maximum estimated gRNA effect magnitude for each target locus is given as a gray dashed line. Nucleosome occupancy (red line), and smoothed ATAC-seq read density (green line) relative to the region maximum are scaled to the maximum effect magnitude. **c** gRNA effects (y-axis) are plotted (black dots) against nucleosome occupancy score (x-axis, left) and ATAC-seq read density (x-axis, right). The median of gRNA effects in windows of 0.25, overlapping by 0.125, is indicated by the circular blue markers. The blue bars show the first and third quartiles. The Spearman correlation for the relationship with nucleosome density is 0.34, *P* value = 9.6 × 10^-12^. The Spearman correlation for the relationship with normalized ATAC-Seq is -0.35, *P* value = 2.2 × 10^-12^

As illustrated above, different gRNAs directed against the same gene can have a range of efficacies (Fig. 2a-d). We tested whether the effective target window reported for CRISPRi in human cell lines contributes to this variability [15]. To do so, we created a library of 383 full-length guides targeting -500 bp to +500 bp of the TSS region of five genes (broad_tiling library), and challenged this library with four chemical compounds. Combining these data with those from the gene_tiling20 library above, we found that the median guide effect was maximal in the window of -200 bp to TSS, while guides downstream of the TSS, or further than 300 bp upstream of the TSS were less effective (Fig. 5a). Effective repression outside the -200 bp to TSS window did occur, but less frequently.

The asymmetry of guide effectiveness around the TSS, and the variability between closely positioned guides indicate that absolute distance to the TSS is not the only determinant of efficacy. As yeast promoters are known to be nucleosome-free, with strictly positioned nucleosomes following the TSS [31–33], we hypothesized that chromatin accessibility and nucleosome occupancy play a role in guide efficacy. We extracted nucleosome occupancy and average chromatin accessibility scores from yeast ATAC-seq data [34], and plotted these data with guide effects in Fig. 5b. A positive relationship between chromatin accessibility and gRNA efficacy was most apparent for the *SEC14* and *SSL2* loci. We next systematically quantified the influence of accessibility on guide efficacy. In the TSS -400 bp to TSS +400 bp window, guides targeting nucleosome-free and ATAC-seq accessible regions were more effective (Fig. 5c). The relationship with ATAC-seq read density persists in the typically nucleosome-occupied region of TSS to TSS +400 bp, suggesting that accessibility influences efficacy independently of positioning relative to the TSS (Additional file 2: Figure S7A). Similar results were obtained when our data were compared to other genome-wide nucleosome position data [33] (Additional file 2: Figure S7B and Additional file 15).

We tested a range of additional potential determinants of guide efficacy (Additional file 16). We first considered the sequence context of the target and found no specific base pairs that were significantly correlated with gRNA efficacy (Additional file 2: Figure S8). Next, we used data from Reimand *et al.* [35] to seek transcription factors whose known or inferred presence in the target region is correlated with guide potency. We found a small number of cases where overlap with a transcriptional activator binding site correlated with stronger guide effects (Additional file 17). Finally, we did not observe a strong effect of RNA secondary structure or melting temperature on gRNA efficacy (Additional file 2: Figure S9).

Our results primarily identify position relative to the TSS and chromatin state as important determinants of whether a gRNA will enable robust transcriptional repression by dCas9. For example, 39 % (171/442) of full-length gRNAs (which targeted regions +/-500 bp from the TSS) exhibited effective repression (gRNA effect < -2) in our assay. On the other hand, gRNAs that target the 200 bp region immediately upstream of the TSS and a nucleosome-depleted region, were effective 76 % (59/78) of the time. Even though additional factors may determine whether a specific gRNA will be a strong transcriptional modulator, applying these two criteria will likely improve performance of future gRNA libraries. We have created a webtool (http://lp2.github.io/yeast-crispri/) to enable rapid design of gRNAs for effective CRISPRi in yeast.

## Discussion

We demonstrated that CRISPRi with inducible gRNA expression is a useful and effective tool to repress genes in yeast. In particular, CRISPRi provides a good alternative to other approaches for studying essential genes [36–39]. CRISPRi constructs can be readily transformed into existing knock-out, GFP-tagged, or other collections, thus enabling genome-wide effects of repressing a particular gene to be characterized. We further showed that CRISPRi, paired with complex gRNA libraries, can be used in competitive growth assays for functional and/or chemical genomic screens. Additionally, CRISPRi plasmid libraries can easily be transformed into any number of different strain backgrounds.

Consistent with CRISPRi being specific for the intended target, increased small molecule sensitivity of gene knockdown strains was largely confined to the predicted gene/drug combinations (Fig. 2c, d and Additional file 2: Figure S4B, S4C). We also analyzed our gRNAs for possible sites of off-target binding using ECRISP [40], and found very few sites that could potentially result in a growth defect (by repression of an essential gene), and none in the TSS region of the 20 genes we focused on (Additional file 18). Nevertheless, it is difficult to know if, or to what extent, off-target binding occurred in our experiments. By employing multiple guides directed against the same target however, one can be more confident that phenotypes observed with multiple independent guides are due to repression of the intended target and not off-target repression. Using this strategy, we uncovered and confirmed a novel chemical-genetic interaction, where Erg25 repression results in resistance to the common antifungal drug, fluconazole.

In our yeast data, truncated gRNAs do not greatly reduce mismatch tolerance when used with the dCas9-Mxi1 repressor. This result is inconsistent with the findings from human cell lines using nuclease-proficient Cas9 [20, 22], and implies one of several possibilities. First, truncated gRNAs could be effective in reducing mismatch tolerance in human cells, but not in yeast. Alternatively, mismatched truncated guides may reduce Cas9’s ability to cleave when compared to equivalent mismatched full length guides, but not its ability to bind to target sequences. dCas9-Mxi1 may only need to bind to the target site to induce transcriptional repression. It is therefore possible that nuclease-proficient Cas9 is still recruited to mismatched target sites by truncated gRNAs but is no longer able to cleave its target. Further studies are required to test these hypotheses. Additionally, we observed that truncated gRNAs that are a perfect match to their target are generally less potent than their full-length counterparts. Thus, we found no clear advantage in using truncated guides for CRISPRi in *S. cerevisiae*.

Our results on ideal guide positioning also differ from those found in human cell lines in which the optimal window for CRISPRi was found to be downstream of the TSS in the 5’UTR [15]. In yeast, we found the optimal window to be a 200 bp region immediately upstream of the TSS. While this difference could be due to the different repressors used (Mxi1 vs KRAB), it could also reflect differences in chromatin structure between yeast and mammalian cells [41, 42]. We observed strong and statistically significant links between guide efficacy and nucleosome occupancy, as well as chromatin accessibility. Nucleosome positioning will likely affect gRNA function in other organisms, and thus successful gRNA design is likely to be species- and even locus-specific. Indeed, ChIP-seq analysis of dCas9 binding in mammalian cells has shown that dCas9 is more likely to bind off-target sites in open chromatin regions than in closed chromatin [21]. Our study thus defines simple design rules taking these correlates into account that will increase the likelihood of gRNAs having a potent repressive effect.

## Conclusions

We have established a powerful functional and chemical genomics screening platform using the CRISPR/Cas9 system for targeted transcriptional repression in *S. cerevisiae*. A reference set of chemical-genetic interactions enabled sensitive measurement of gRNA efficacy at multiple loci. Most notably, we found that truncated gRNAs generally exhibited reduced efficacy towards both mismatched and perfectly matched target sequences compared to their full-length counterparts. In addition, we identify nucleosome occupancy as a major determinant of gRNA performance. gRNAs directed to a region between the TSS and 200 bp upstream of the TSS were more likely to be effective. These findings will directly enable library design and genome-wide screening in yeast, and may also inform the application of CRISPRi in other organisms.

## Methods

### Plasmid and strain construction

All primers, strains, and plasmids used in this study are listed in Additional file 19. All chemical compounds used in this study are listed in Additional file 8. Molecular cloning was done with Gibson Assembly as outlined in Gibson *et al.* [43]. *E. coli* minipreps were performed with QIAprep Spin Minipreps (Qiagen). Preparation of competent *E. coli* DH5α and transformation used Zymo Mix & Go *E. coli* Transformation reagents and Zymo Broth. Hifi Hotstart (Kapa Biosystems), Q5 (NEB) and Phusion Hot Start Flex (Thermo Scientific) high fidelity polymerases were used for PCRs. Primers and single gRNA oligonucleotides were ordered from IDT. gRNA oligo libraries were ordered from Custom Array. DpnI treatment was used to remove template plasmids in PCRs that were followed by Gibson Assembly. Benchling.com DNA editing software was used for plasmid design. Individual constructs (not libraries) were sequenced by Sanger Sequencing (Sequetech).

To build the dCas9 repressor, we first modified pRS414-Tef1-Cas9-Cyc1t obtained from addgene [1] to introduce the D10A and H840A mutations to produce dCas9. We also fused a nuclear localization signal to the N terminus of dCas9. The human Mxi1 domain and linker from [6] was then fused to the C terminal of dCas9.

We built our single plasmid system in the yeast pRS414 and pRS416 Cen/ARS plasmids containing the Trp1 and Ura3 markers, respectively. First, we introduced an engineered Tet inducible pRPR1 PolIII promoter [7, 24, 44], NotI site, and gRNA sequence, as well as the Tet repressor (TetR) gene under control of the *GPM1* promoter and terminator into pRS414-Tef1-NLS-dCas9-Mxi1-Cyc1 at the PciI site adjacent to the ori using Gibson Assembly. These vectors are referred to as pRS41XgT. We then PCRed the gRNA and TetR and cloned them into pRS416 digested with PciI along with a bridging oligo to correct the PciI site cut in Ura3. Next we PCRed and cloned the Tef1-NLS-dCas9-Mxi1-Cyc1 section of the plasmid into this vector. We are providing our tet-inducible CRISPRi plasmid on AddGene for other investigators to study their questions of interest.

gRNA oligos were amplified with extender oligos that produced 40 bp overlaps on either side of the region of target complementarity, and then cloned into the NotI site with Gibson Assembly. The same protocol was applied both to individual oligos and libraries of oligos. These were then transformed into DH5α cells and plated on LB-agar containing carbenicillin. For individual clones, single colonies were obtained and screened by colony PCR and Sanger sequencing. Correct colonies were cultured and plasmids extracted. For libraries, all colonies were washed off plates with LB-Carb liquid and then minipreped.

Competent *S. cerevisiae* (strain BY4741) were prepared either by standard lithium acetate transformation protocols or using Frozen-EZ Yeast Transformation II Kit™ (Zymo Research). Transformed cells were selected on synthetic complete media (SCM) – Ura agar plates. For individual strains, single colonies were selected for additional experiments. For library preparation, all colonies were washed off plates with SCM-Ura liquid media, vortexed, and aliquoted into 25 % glycerol stocks of 3.0 ODs of cells each for later use.

### Growth assays of individual strains

Strains were grown overnight in synthetic complete media lacking uracil (SCM-Ura). Growth assays were performed in 96 well NUNC flat bottom plates in 100 μL SCM–Ura cultures. Starting OD_600_ was either OD_600_ 0.01 or 0.03, but was consistent within individual experiments. Growth rates were determined by measuring the OD_600_ approximately every 15 min for at least 80 cycles at 30 °C in TECAN sunrise or GENios plate readers. Drugs were dissolved in DMSO and dispensed to plates using an HP D300 Digital Dispenser (Tecan). The growth rate of a strain was calculated as follows:(1) the first 10 OD readings were averaged and subtracted from all OD readings of the corresponding curve in order to set the baseline of the growth curve to zero; (2) the area under the curve (AUC) was then calculated as the sum of all OD readings. ‘Relative growth’ was calculated as previously described [45], and as follows: (AUC_condition_ – AUC_control_)/AUC_control_; where AUC_control_ represents the growth rate of the reference condition that was assayed on the same microtiter plate.

### qPCRs

For qPCR experiments, strains were typically cultured in SCM–Ura media overnight, diluted to an OD/mL of 0.15 in the presence (or absence) of 250 ng/mL ATC, grown further, and samples collected at the times indicated. For the ATc removal time course, cells were washed five times with sterile water to remove any residual ATc. RNA was extracted from samples using the Ambion RiboPure™ RNA Purification Kit for yeast (Life Technologies), or the Quick RNA Kit (Zymo Research). RNA was converted to cDNA using the High-Capacity RNA-to-cDNA™ Kit (Life Technologies). This cDNA was diluted 1:10 and then used for SYBR qPCR. qPCR primers were designed using primer3 to give products of approximately 75-150 bases in length (Additional file 19). Real time/qPCR was performed using SYBR® Green PCR Master Mix (Life Technologies) and the Applied Biosystems 7900HT Fast Real-Time PCR System running SDS V2.3. The gRNAs used for these experiments are listed in Additional file 19. Log2 fold change relative to a reference condition was calculated as the negative delta delta Ct (-DDCt) as follows: DDCt = ((average Ct)_gene_-(average Ct)_control gene_) in test condition – ((average Ct)_gene_ – (average Ct)_control gene_) in reference condition. Average Ct values were typically calculated from four replicates. Standard deviation (StdDev) was calculated as the square root of ((StdDev of Ct_gene_)^2^ + (StdDev of Ct_control__gene_)^2^) as measured in the test condition.

### Library design

The ‘gene tiling’ libraries were designed to a window of 150 bp upstream of the TSS to 50 bp into the ORF. TSS were specified as the most common transcript start position from transcript isoform profiling data [46], or inferred to be a fixed distance of 27 bp upstream of the start codon based in part on previous results [47]. Excluding 41/442 guides targeting genes without transcript isoform profiling support for the TSS, did not affect the results (Additional file 2: Figure S10). A full list of genes examined are available in Additional file 8. Guides were designed both to the template strand and non-template strand. For each of these guides we designed versions containing 18 nt and 20 nt of target complementarity (gene_tiling18 and gene_tiling20, respectively). Even though gRNAs were designed for *CDC12*, *ERO1*, and *RPO21*, small molecule inhibitors specific to these genes were not assayed. For five of the genes (*ERG11*, *ERG25*, *SEC14*, *CRG1*, and *SSL2*) we designed all possible full-length guides within a window of 500 bp up- and downstream of the TSS (broad_tiling).The mutant library was designed by taking the sequences for four gRNAs we had previously shown to be functional and making a random single base change for all positions in the seed sequence (1-10) as well as in positions 11, 12, 14, and 16. For each guide, we synthesized both an 18 and 20 nt version (mutant18 and mutant20, respectively). We used ECRISP version 4.2 to look for potential off-target binding sites in the yeast genome, allowing for up to two mismatches.

### Competitive growth assays

Prior to setting up experiments, aliquots of a library were recovered in YPD media for 4 h, and then diluted appropriately for the experiments. Yeast culturing and sample collection was performed using a cell-screening platform that integrates temperature-controlled absorbance plate readers, plate coolers, and a liquid handling robot. Briefly, 700 μL yeast cultures were grown (+/- a drug listed in Additional file 8, and +/- ATc) in 48 well plates at 30 °C with orbital shaking in Infinite plate readers (Tecan). To maintain cultures in log phase over many doublings, 23 μL of the culture was removed when it reached an OD of 0.76, added to a well containing 700 μL of media, and then allowed to grow further. After three such dilutions, 600 μL of the culture was collected and saved to a 4 °C cooling station (Torrey Pines) when it reached an OD of 0.76. This amounted to approximately 20 culture doublings from the beginning of the experiment. Pipetting events were triggered automatically by Pegasus Software and performed by a Freedom EVO workstation (Tecan).

A key parameter in this protocol is the extent to which a drug inhibits growth of the pool. In general, drug concentrations that inhibit growth by approximately 20 % are best for identifying chemical-genetic interactions and yielding reproducible results. If a drug was observed to inhibit the pool’s growth too strongly (for example, by >50 %), the experiment was repeated using a lower drug concentration.

After sample collection, yeast plasmids were purified using the Zymoprep Yeast Plasmid Miniprep II kit (Zymo Research). Purified plasmids were used as a template for PCR with barcoded up- and down-sequencing primers that produce a double index to uniquely identify each sample. PCR products were confirmed by agarose gel electrophoresis. After PCR, samples were combined and bead cleaned with Thermo Scientific™ Sera-Mag Speed Beads Carboxylate-Modified particles. Sequencing was performed using Illumina MiSeq.

### Metabolite extraction and GCMS analysis

Our previously described methods were used for metabolite measurement [48]. In brief, yeast pellets of six biological replicates were homogenized in 1× PBS buffer with 0.5 mg of 0.5 mm glass beads/tube by vortexing for a total of 6 min. Every 2 min between vortexing, the tubes were returned back to ice. The homogenized mixture was extracted by Folch method [49]. The lower phase of the chloroform:methanol:water mixture, containing the sterol metabolites extracted from the yeast cell pellet, was collected and dried in a Speedvac. The samples were derivatized by MSTFA + 1 % TMCS and analyzed by Agilent 7200 series GC/Q-TOF. The sterols were separated on HP5-MS UI column (30 m, 0.25 mm i.d, 0.25 μm film thickness) at split ratio 20:1 using helium as carrier gas at 1 mL/min. The oven temperature program was as follows: 60 °C held for 1 min, then oven temperature was ramped at 10 °C/min to 325 °C where it was held for 3.5 min. Data were collected at acquisition rate of 5 Hz in both profile and centroid modes. Qualitative and quantitative analysis was performed using Agilent MassHunter Workstation.

Ergosterol, lansterol, and methoxyamine hydrochloride were purchased from Sigma. HPLC grade methanol, chloroform, and water were from Honeywell Burdick and Jackson. The derivatization reagent MSTFA (N-methyl-N-trimethylsilytrifluoroacetamide) with 1 % TMCS (trimethylchlorosilane) was from Thermo Fisher Scientific (Bellefonte, PA, USA).

### Sequence data analysis

We used a combination of established tools and custom python pipelines to quantify gRNA effects from the sequence data. First, we created a synthetic reference chromosome sequence for each of the expected amplicons. The synthetic reference included Illumina adaptors, library barcode, PCR amplification priming region, PCR barcode, and the gRNA region complementary to the target. As the forward and reverse reads were expected to overlap given the sequencing read length, and average 190 bp amplicon length, we merged the reads using PEAR [50] version 0.9.4 with default parameters. The resulting FastQ file was mapped against the created synthetic reference using BWA [51] version 0.6.1-r104 with command line ‘bwa index [reference]; bwa aln -n3 -o3 -e1 -l22 [reference] [fastq] > [aln]; bwa samse -f [out] [reference] [aln] [fastq]’. This parameter setting allows for three mismatches, three gaps, one large gap, and short 22 bp seed sequences. For each of the expected amplicons, we counted the number of perfect matches (flag NM:i:0) from the resulting alignments (Additional file 9, column ‘Count_perfect’) that were used in subsequent analyses.

### Guide fitness calculation

We quantified fitness *f* for guide *i* in pool *j*, condition *k* as the relative growth rate *f*_*ijk*_. For a guide with *c*_*ijk*_ counts in condition *k*, we calculated the log_2_-scale median centered counts *l*_*ijk*_ = log_2_(*c*_*ijk*_ + 0.5) - median_*i’* in pool *j*_(log_2_(*c*_*i’jk*_ + 0.5)). As any such count statistics are highly variable at low abundances, it is important to also record the confidences of this value. We calculated the empirical variance estimate of *l*_*ijk*_ by resampling reads given the total library size of *C*_*jk*_ reads, and *N*_*j*_ guides in pool *j* condition *k*. To do so, we inferred the posterior read frequency in the pool as Gamma(*c*_*ijk*_ + 0.5, *C*_*jk*_ + 0.5*N*_*j*_), sampled 1,000 observations of read counts, calculated the log-scale median centered count for each, and used the variance *s*^*2*^_*ijk*_ of the simulated values as a variance estimate for the log scale counts. In the following, we thus model guide fitness as *f*_*ijk*_ ~ Normal(*l*_*ijk*_, *s*^*2*^_*ijk*_).

### ATc-induced fold change

To estimate the guide effect on growth, we calculated the ATc-induced fold change (*A*). For conditions *k +* and *k-* (with and without ATc, respectively), we infer the ATc-induced fold change *A*_*ijk*_ on guide *i* in pool *j* as the difference in the fitness between the cultures with and without ATc, *A*_*ijk*_ *= f*_*ijk+*_*- f*_*ijk-*_ ~ Normal(*l*_*ijk+*_*- l*_*ijk-*_*, s*^*2*^_*ijk+*_ + *s*^*2*^_*ijk-*_). For the control condition (1 % DMSO), we had eight replicate experiments for the tiling pools, and three replicate experiments for 20 μM fluconazole. We combined the *R* replicates *k*_*1*_,…,*k*_*R*_ in a natural way to obtain the variance var(*A*_*ijk*_) = 1/(1/var*(A*_*ijk1*_*)* + … + 1/*var(A*_*ijkR*_*)),* and mean < *A*_*ijk*_ *> =* var*(A*_*ijk*_) * (<A_*ijk1*_*>/*var*(A*_*ijk1*_*) + … + <A*_*ijkR*_*>/*var*(A*_*ijkR*_*)).* The combined estimate across replicates is then *A*_*ijk*_ ~ Normal(<*A*_*ijk*_*>,* var(*A*_*ijk*_)). gRNAs with fewer than 30 reads following growth in the minus ATc control condition were excluded from this analysis, as their effect size estimates had large variance across conditions.

The ATc-induced fold change (*A*) of a gRNA, calculated following growth in the presence of its specific partner reference compound (Additional file 8), was defined as the ‘gRNA effect’ or ‘guide effect’ and used in Figs. 4, 5c, Additional file 2: Figure S6, and S7. In cases where we tested multiple concentrations of a reference compound, we selected data from one concentration (these are indicated in Additional file 8). For the plots in Fig. 5a, b, and Additional file 2: Figure S9, we first restrict *A* values to have a maximum of 0, and then take the absolute value of this. We define this as the ‘gRNA effect magnitude’. This calculation is based on the reasonable assumption that repression of the target gene can only result in drug-sensitivity (that is, a negative ATc-induced fold change), and that any apparent resistance (that is, positive ATc-induced fold change) is a result of technical noise in a poorly functional gRNA.

### Drug-specific effect estimation

To estimate the drug-specific effect *D* for guide *i* in pool *j* and drug *k*, we contrasted the guide fitness with and without drug when the system was induced with ATc, that is, *D*_*ijk*_ *= f*_*ijk+*_*- f*_*ij0+*_ *~* Normal*(l*_*ijk+*_*- l*_*ij0+*_*, s*^*2*^_*ijk+*_ + *s*^*2*^_*ij0+*_*)*, where condition *k* = 0 is the 1 % DMSO control, and its parameters are for the distribution inferred from the eight replicates. gRNAs having fewer than 30 reads following growth in the presence of ATc, but without additional chemical compound, were excluded from this analysis, as their effect size estimates had large variance across conditions.

### Guide melting temperature calculation

We used ViennaRNA package 2.0 (RNAfold version 2.1.9 ViennaRNA Package 2.0) [52] to calculate RNA folding and duplex formation energies for the entire targeting region, as well as the eight nucleotide seed, and the oligotm [53] library, both with default parameters.

### ATAC-seq and nucleosome data

We downloaded the nucleosome occupancy and ATAC-seq insertion data from Schep *et al.* [34] in bigWig format, and converted it to wig using UCSC utilities (./bigWigToWig < input > < output > -chrom = < chromosome > -start = < TSS-1000 > -end = < TSS + 1000 >). For each nucleotide, we used the per-base.wig output as the measure of nucleosome occupancy, and the average ATAC-seq insert count in 51 base window centered on the base as the ATAC-seq signal. Spearman correlation was calculated using the spearmanr function in the scipy.stats python package.

We also compared the efficacy of gRNAs targeting *ERG11*, *ERG25*, *SEC14*, and *SSL2* to yeast nucleosome occupancy measurements reported by Lee *et al.* [33]. Based on previous exonuclease footprinting experiments [54], we first defined a genomic region predicted to be occupied by dCas9 upon successful base pairing with the gRNA. This region (or ‘window’) consisted of the genomic target sequence recognized by the gRNA, plus three bases on either end. Window coordinates were defined based on the February 2006 SGD genome build as these data were used in the analysis by Lee *et al.* [33]. We downloaded log2 ratios representing the relative hybridization signal of total genomic DNA to nucleosomal DNA. These measurements were made using a 4 bp resolution tiling array of the yeast genome, and therefore, each gRNA has 6 or 7 log2 values within its window. These values were averaged to generate the ‘Nucleosome Occupancy’ values plotted for each gRNA in Additional file 2: Figure S7B, which were compared to the effects of gRNAs for *ERG11*, *ERG25*, *SEC14*, and *SSL2*; R and *P* values (Spearman correlation) were calculated in Spotfire (Perkin Elmer).

### Target sequence context

We considered the region of 20 bp upstream of the PAM to 20 bp downstream of the end of the target sequence. For each site in this region, and each of A, C, G, T bases, we calculated the relative gRNA effect in control condition for all the guides whose target sequence has that base at the considered site. Relative gRNA effect was calculated by dividing the gRNA effect for each guide by the maximum gRNA effect constrained to be between 0 and -6 for each control drug/gene set. We calculated the *P* value of the median effect as the fraction of 10,000 random samples of same number of overlapping guides that have at least as large median effect. We also report *P* values Bonferroni-corrected for the number of tests (4 bases × 63 sites = 252 tests).

### Overlap with transcription factor binding sites

We downloaded data from Reimand *et al.* [35], as used in Zaugg and Luscombe [31]. We considered three levels of overlap - sites overlapping middle of the specificity-determining sequence, middle 10 bases of the sequence, and any part of the sequence. For each factor, and each level of overlap, we calculated the mean guide effect magnitude in control condition of all the overlapping guides in gene_tiling20 and broad_tiling guide sets. For each level, we only considered factors that overlapped at least 10 guides. We calculated the *P* value of the median effect as the fraction of 10,000 random samples of same number of overlapping guides that have at least as large mean effect. We also report *P* values Bonferroni-corrected for the number of tests.

### Availability of supporting data

The data sets supporting the results of this article are available in the Gene Expression Omnibus (GEO) repository, GSE71490; http://www.ncbi.nlm.nih.gov/geo/query/acc.cgi?acc=GSE71490.

### Ethics approval

This study did not require ethics approval.

## Additional files

Additional file 1: Raw growth data measurements for Fig. 1c. (XLSX 807 kb) Additional file 2: Supplemental figures. (PDF 16018 kb) Additional file 3: Raw data for qPCR. (XLSX 38 kb) Additional file 4: List of gRNAs, their target location, and target strand (template or non-template). (XLSX 81 kb) Additional file 5: List of sequenced samples, their respective barcodes, and other metadata. (XLSX 18 kb) Additional file 6: Counts and analysis for eight ‘no-drug’ replicates. (XLSX 211 kb) Additional file 7: Counts and analysis of three replicates in 20 μM fluconazole. (XLSX 150 kb) Additional file 8: The small molecule/gene combinations constituting the reference chemical-genetic interactions used in this study. (XLSX 11 kb) Additional file 9: Sequence read information and raw counts from MiSeq data. (XLSX 3070 kb) Additional file 10: ATc effects (A), drug effects (D), and no-drug ATc effects (A0) for each gRNA in every tested condition. (XLSX 2152 kb) Additional file 11: Drug-specific effects (D) for all drugs tested for each gRNA. (XLSX 416 kb) Additional file 12: Average drug-specific effects for each guide set (gRNAs directed against the same gene). (XLSX 27 kb) Additional file 13: Raw growth data for Fig. 3. (XLSX 28 kb) Additional file 14: GCMS measurements of ergosterol intermediates in yeast treated with 1181-0519. (XLS 50 kb) Additional file 15: Nucleosome occupancy scores of gRNA target regions based on tiling array analysis. (XLSX 49 kb) Additional file 16: A range of additional potential determinants of guide effect. (XLSX 2213 kb) Additional file 17: Number and significance of transcription factor site overlaps with positive control gRNA target sequences. (XLSX 10 kb) Additional file 18: Analysis of potential off-target binding sites. (XLSX 36 kb) Additional file 19: Primers, strains, and plasmids used in this study. (XLSX 96 kb)

## Abbreviations

ATc: Anhydrotetracycline
AUC: area under the curve
CRISPR: clustered regularly interspaced palindromic repeats
CRISPRi: CRISPR interference
DMSO: dimethyl sulfoxide
gRNA: guide RNA
MoA: mechanism-of-action
nt: nucleotide
ORF: open reading frame
PAM: protospacer adjacent motif
TSS: transcription start site

## References

[1] DiCarlo JE, Norville JE, Mali P, Rios X, Aach J, Church GM (2013). Genome engineering in Saccharomyces cerevisiae using CRISPR-Cas systems. Nucleic Acids Res.

[2] Mali P, Yang L, Esvelt KM, Aach J, Guell M, DiCarlo JE (2013). RNA-guided human genome engineering via Cas9. Science.

[3] Jinek M, Chylinski K, Fonfara I, Hauer M, Doudna JA, Charpentier E (2012). A programmable dual-RNA-guided DNA endonuclease in adaptive bacterial immunity. Science.

[4] Cong L, Ran FA, Cox D, Lin S, Barretto R, Habib N (2013). Multiplex genome engineering using CRISPR/Cas systems. Science.

[5] Qi LS, Larson MH, Gilbert LA, Doudna JA, Weissman JS, Arkin AP (2013). Resource repurposing CRISPR as an RNA-guided platform for sequence-specific control of gene expression. Cell.

[6] Gilbert LA, Larson MH, Morsut L, Liu Z, Brar GA, Torres SE (2013). CRISPR-mediated modular RNA-guided regulation of transcription in eukaryotes. Cell.

[7] Farzadfard F, Perli SD, Lu TK (2013). Tunable and multifunctional eukaryotic transcription factors based on CRISPR/Cas. ACS Synth Biol.

[8] Perez-Pinera P, Kocak DD, Vockley CM, Adler AF, Kabadi AM, Polstein LR (2013). Brief communications RNA-guided gene activation by CRISPR- Cas9 – based transcription factors. Nat Methods.

[9] Maeder ML, Linder SJ, Cascio VM, Fu Y, Ho QH, Joung JK (2013). CRISPR RNA-guided activation of endogenous human genes. Nat Methods.

[10] Mali P, Aach J, Stranges PB, Esvelt KM, Moosburner M, Kosuri S (2013). CAS9 transcriptional activators for target specificity screening and paired nickases for cooperative genome engineering. Nat Biotechnol.

[11] Shalem O, Sanjana NE, Hartenian E, Shi X, Scott DA, Mikkelsen TS (2014). Genome-scale CRISPR-Cas9 knockout screening in human cells. Science.

[12] Wang T, Wei JJ, Sabatini DM, Lander ES (2014). Genetic screens in human cells using the CRISPR-Cas9 system. Science.

[13] Zhou Y, Zhu S, Cai C, Yuan P, Li C, Huang Y (2014). High-throughput screening of a CRISPR/Cas9 library for functional genomics in human cells. Nature.

[14] Koike-Yusa H, Li Y, Tan E-P, Velasco-Herrera MDC, Yusa K (2014). Genome-wide recessive genetic screening in mammalian cells with a lentiviral CRISPR-guide RNA library. Nat Biotechnol.

[15] Gilbert LA, Horlbeck MA, Adamson B, Villalta JE, Chen Y, Whitehead EH (2014). Genome-scale CRISPR-mediated control of gene repression and activation. Cell.

[16] Konermann S, Brigham MD, Trevino AE, Joung J, Abudayyeh OO, Barcena C (2014). Genome-scale transcriptional activation by an engineered CRISPR-Cas9 complex. Nature.

[17] Fu BXH, Hansen LL, Artiles KL, Nonet ML, Fire AZ (2014). Landscape of target: guide homology effects on Cas9-mediated cleavage. Nucleic Acids Res.

[18] Hsu PD, Scott DA, Weinstein JA, Ran FA, Konermann S, Agarwala V (2013). DNA targeting specificity of RNA-guided Cas9 nucleases. Nat Biotechnol.

[19] Fu Y, Foden JA, Khayter C, Maeder ML, Reyon D, Joung JK (2013). High-frequency off-target mutagenesis induced by CRISPR-Cas nucleases in human cells. Nat Biotechnol.

[20] Tsai SQ, Zheng Z, Nguyen NT, Liebers M, Topkar VV, Thapar V (2014). GUIDE-seq enables genome-wide profiling of off-target cleavage by CRISPR-Cas nucleases. Nat Biotechnol.

[21] Wu X, Scott DA, Kriz AJ, Chiu AC, Hsu PD, Dadon DB (2014). Genome-wide binding of the CRISPR endonuclease Cas9 in mammalian cells. Nat Biotechnol.

[22] Fu Y, Sander JD, Reyon D, Cascio VM, Joung JK (2014). Improving CRISPR-Cas nuclease specificity using truncated guide RNAs. Nat Biotechnol.

[23] Sikorski RS, Hieter P (1989). A system of shuttle vectors and yeast host strains designed for efficient manipulation of DNA in Saccharomyces cerevisiae. Genetics.

[24] Bak G, Hwang SW, Ko Y, Lee J, Kim Y, Kim K (2010). On-off controllable RNA hybrid expression vector for yeast three-hybrid system. BMB Rep.

[25] Hoon S, Smith AM, Wallace IM, Suresh S, Miranda M, Fung E (2008). An integrated platform of genomic assays reveals small-molecule bioactivities. Nat Chem Biol.

[26] Lee AY, St Onge RP, Proctor MJ, Wallace IM, Nile AH, Spagnuolo PA (2014). Mapping the cellular response to small molecules using chemogenomic fitness signatures. Science.

[27] Winzeler EA, Shoemaker DD, Astromoff A, Liang H, Anderson K, Andre B (1999). Functional characterization of the S. cerevisiae genome by gene deletion and parallel analysis. Science.

[28] Giaever G, Chu AM, Ni L, Connelly C, Riles L, Véronneau S (2002). Functional profiling of the Saccharomyces cerevisiae genome. Nature.

[29] Smith AM, Heisler LE, St Onge RP, Farias-Hesson E, Wallace IM, Bodeau J (2010). Highly-multiplexed barcode sequencing: an efficient method for parallel analysis of pooled samples. Nucleic Acids Res.

[30] Smith AM, Heisler LE, Mellor J, Kaper F, Thompson MJ, Chee M (2009). Quantitative phenotyping via deep barcode sequencing. Genome Res.

[31] Zaugg JB, Luscombe NM (2012). A genomic model of condition-specific nucleosome behavior explains transcriptional activity in yeast. Genome Res.

[32] Yuan G-C, Liu Y-J, Dion MF, Slack MD, Wu LF, Altschuler SJ (2005). Genome-scale identification of nucleosome positions in S. cerevisiae. Science.

[33] Lee W, Tillo D, Bray N, Morse RH, Davis RW, Hughes TR (2007). A high-resolution atlas of nucleosome occupancy in yeast. Nat Genet.

[34] Schep A, Buenrostro JD, Denny SK, Schwartz K, Sherlock G, Greenleaf WJ (2015). Structured nucleosome fingerprints enable high-resolution mapping of chromatin architecture within regulatory regions. Cold Spring Harbor Labs J.

[35] Reimand J, Vaquerizas JM, Todd AE, Vilo J, Luscombe NM (2010). Comprehensive reanalysis of transcription factor knockout expression data in Saccharomyces cerevisiae reveals many new targets. Nucleic Acids Res.

[36] Li Z, Vizeacoumar FJ, Bahr S, Li J, Warringer J, Vizeacoumar FS (2011). Systematic exploration of essential yeast gene function with temperature-sensitive mutants. Nat Biotechnol.

[37] Davierwala AP, Haynes J, Li Z, Brost RL, Robinson MD, Yu L (2005). The synthetic genetic interaction spectrum of essential genes. Nat Genet.

[38] Breslow DK, Cameron DM, Collins SR, Schuldiner M, Stewart-Ornstein J, Newman HW (2008). A comprehensive strategy enabling high-resolution functional analysis of the yeast genome. Nat Methods.

[39] Yan Z, Costanzo M, Heisler LE, Paw J, Kaper F, Andrews BJ (2008). Yeast Barcoders: a chemogenomic application of a universal donor-strain collection carrying bar-code identifiers. Nat Methods.

[40] Heigwer F, Kerr G, Boutros M (2014). E-CRISP: fast CRISPR target site identification. Nat Methods.

[41] Rando OJ, Chang HY (2009). Genome-wide views of chromatin structure. Annu Rev Biochem.

[42] Rando OJ, Winston F (2012). Chromatin and transcription in yeast. Genetics.

[43] Gibson DG, Young L, Chuang R-Y, Venter JC, Hutchison CA, Smith HO (2009). Enzymatic assembly of DNA molecules up to several hundred kilobases. Nat Methods.

[44] Lee JY, Evans CF, Engelke DR (1991). Expression of RNase P RNA in Saccharomyces cerevisiae is controlled by an unusual RNA polymerase III promoter. Proc Natl Acad Sci USA.

[45] Schlecht U, Miranda M, Suresh S, Davis RW, St Onge RP (2012). Multiplex assay for condition-dependent changes in protein-protein interactions. Proc Natl Acad Sci U S A.

[46] Pelechano V, Wei W, Steinmetz LM (2013). Extensive transcriptional heterogeneity revealed by isoform profiling. Nature.

[47] Zhang Z, Dietrich FS (2005). Mapping of transcription start sites in Saccharomyces cerevisiae using 5’ SAGE. Nucleic Acids Res.

[48] Wu M, Zheng M, Zhang W, Suresh S, Schlecht U, Fitch WL (2012). Identification of drug targets by chemogenomic and metabolomic profiling in yeast. Pharmacogenet Genomics.

[49] Folch J, Lees M, Sloane Stanley GH (1957). A simple method for the isolation and purification of total lipides from animal tissues. J Biol Chem.

[50] Zhang J, Kobert K, Flouri T, Stamatakis A (2014). PEAR: a fast and accurate Illumina Paired-End reAd mergeR. Bioinformatics.

[51] Li H, Durbin R (2009). Fast and accurate short read alignment with Burrows-Wheeler transform. Bioinformatics.

[52] Lorenz R, Bernhart SH, Höner Zu Siederdissen C, Tafer H, Flamm C, Stadler PF (2011). ViennaRNA Package 2.0. Algorithms Mol Biol.

[53] Rozen S, Skaletsky H (2000). Primer3 on the WWW for general users and for biologist programmers. Methods Mol Biol.

[54] Jinek M, Jiang F, Taylor DW, Sternberg SH, Kaya E, Ma E (2014). Structures of Cas9 endonucleases reveal RNA-mediated conformational activation. Science.

[55] Kelly SL, Lamb DC, Corran AJ, Baldwin BC, Kelly DE (1995). Mode of action and resistance to azole antifungals associated with the formation of 14 alpha-methylergosta-8,24(28)-dien-3 beta,6 alpha-diol. Biochem Biophys Res Commun.

[56] Vale-Silva LA, Coste AT, Ischer F, Parker JE, Kelly SL, Pinto E (2012). Azole resistance by loss of function of the sterol Δ^5^,^6^-desaturase gene (ERG3) in Candida albicans does not necessarily decrease virulence. Antimicrob Agents Chemother.

