# Supplementary material for: Quantitative CRISPR interference screens in yeast identify chemical-genetic interactions and new rules for guide RNA design
Source: Genome Biol. 2016 Mar 8;17:45. doi: 10.1186/s13059-016-0900-9 (PMC4784398; doi:10.1186/s13059-016-0900-9)
Supplement: Additional file 2: — Supplemental figures. (PDF 16018 kb) [file 13059_2016_900_MOESM2_ESM.pdf]

Supplemental Figure Legends

Figure S1. Characterization of single-plasmid system for CRISPRi in yeast. A) Transformants expressing gRNAs directed against *CRG1, ERG11, ERG25,* and *SEC14* (see legend), were grown in the presence of a specific small molecule (*i.e.* cantharidin (10μM), fluconazole (25μM), 1181-0519 (20μM), and 4130-1276 (2.5μM), respectively), and in increasing concentrations of ATc (x-axis). Growth relative to the 'no-ATc' control is indicated on the y-axis (see Methods). B - E) SYBR qPCR results examining the effects various gRNAs on gene expression. Each plot shows expression change (log2 fold change) relative to a control condition. All plots use *ACT1* as a reference gene, with the exception of B which uses *UBC6*. Error bars represent the standard deviation. Raw data are available in **Additional file 3**. B) Strains expressing gRNAs targeting *CRG1*, *ERG11*, or *ERG25* (plus empty vector control) were cultured in the presence or absence of ATc (see legend). Expression relative to the empty vector control in the absence of ATc (EV-Mxi1-ATc), is measured for each gene and the gRNA (x-axis). C) *RBD2* expression changes following induction of the P20 strain (*RBD2* gRNA) with ATc. Log2 fold change (relative to the time zero control) is plotted on y-axis, for different time points following ATc addition on the x-axis. D) Similar to C, only for P12 (*ERG25* gRNA). Both *ERG11* and *ERG25* expression is plotted. E) *CRG1* expression changes following removal of ATc from an induced culture. The P141 strain (*CRG1* gRNA) was grown overnight in the presence or absence of ATc. Cells from induced culture (+ATc) were centrifuged and washed several times with water, and then resuspended in media without ATc. Samples were collected at various times following removal of ATc (x-axis). Expression relative to that of the overnight culture grown without ATc is plotted on the y-axis.

Figure S2. Schematic of the workflow for parallel analysis of CRIPSRi-induced fitness defects in pooled cultures. Oligos were synthesized by oligo array synthesis by Custom Array. These were PCR-amplified and Gibson Assembled into NotI digested pRS416gT-Mxi1 and transformed into *E. coli*. The plasmids were purified and transformed into yeast. Once the yeast pool was built, experiments were conducted in which pools were grown in inducing (+ATc) and non-inducing conditions (-ATc), in the presence (or absence) of different small molecules (aka "drugs"). After multiple generations of growth in these conditions, yeast plasmids were extracted and the gRNA region complementary to its target was PCR-amplified and sequenced on a MiSeq. Counts of each gRNA were compared between different conditions. The table summarizes the different growth conditions compared in this study, and the information that each comparison can provide.

Figure S3. A) Plots comparing the gRNA sequence counts (log2-transformed) from 8 +ATc replicates and 8 –ATc replicates. B) Plots comparing the log2-transformed gRNA sequence counts for 3 +ATc replicates and 3 –ATc replicates in fluconazole.

Figure S4. Parallel analysis of CRIPSRi-induced fitness defects in pooled cultures. Drug/gene pairs representing reference chemical-genetic interactions are shown in grey. (A) Effect of gRNA expression on growth in the presence of drug (indicated above each plot) as in Figure 2A. Median-adjusted ATc effect (***A***) is plotted on the y-axis. Each point represents a unique gRNA directed against one of 20 different target genes. gRNAs are color-coded and arranged alphabetically on the x-axis by target gene. B) Similar to (A), only the y-axis quantifies drug-specific effects as in Figure 2C. Large black dots represent the mean for each gene, and are colored red if >1 or if <-1. C) Drug-specific growth defects for each gRNA set (group of guides directed against the same gene; indicated above each plot) in 25 different drug conditions (arranged on the x-axis), as in Figure 2D. Points are color-coded by condition. Large black dots represent the mean in each drug condition, and are colored red if >1 or if <-1.

Figure S5. Effect of 1181-0519 on ergosterol metabolite levels in yeast. A simplified schematic of the ergosterol synthesis pathway is illustrated. The abundance of six metabolites from yeast treated with 1181-0519 (blue bars), or DMSO control (black bars) is shown in the bar plots. Error bars represent SEM. Consistent with chemical inhibition of Erg25 by 1181-0519, the abundance of immediate upstream metabolite of Erg25, 4,4-dimethyl-5α-cholesta-8,24-dien-3b-ol (aka 4, 4-dimethylzymosterol) increased by 6.2 fold in the presence of 1181-0519 with a p-value of $2.9x10^{-4}$, while the abundance of downstream metabolite zymosterol decreased by 6.0 fold with a p-value of $1.2x10^{-5}$. The abundance of ergosterol decreased by 1.3 fold with a p-value of $4.1x10^{-3}$.

Figure S6. Quantitative comparison of full-length and truncated gRNAs. Heatmaps illustrating the ATc effects measured for gRNAs containing different mismatches to the target sequence. Full-length (20 nt of target complementarity) and truncated (18 nt of target complementarity) gRNAs are arranged by target gene on the y-axis. The mismatch position of each gRNA relative to the PAM is indicated on the x-axis (gRNAs matching the target sequence perfectly are on the far left). Missing values are indicated with an X. Each panel represents results for a different drug (indicated above).

Figure S7. The effect of nucleosome occupancy and chromatin accessibility on gRNA efficacy. A) As in Figure 5C, guide effect relative to nucleosome occupancy and chromatin accessibility, for gRNAs targeting 0 to 400 bp downstream of the TSS. The median of gRNA effects in windows of 0.25, overlapping by 0.125, is indicated by the circular blue markers. The blue bars show the first and third quartiles. The Spearman correlation for the relationship with nucleosome density is 0.06, p-value = .44. The Spearman correlation for the relationship with normalized ATAC-Seq is -0.31, p-value = $9.3x10^{-6}$. B) Nucleosome occupancy based on previously published measurements[33] (see Methods) is plotted on x-axis versus guide effect (broad tiling library) on the y-axis. Negative guide effect values indicate stronger gRNA-induced growth

defects. Each panel represents a different locus (indicated above each plot). Guide effect values were calculated based on growth in the small molecule specific to each locus. R and p-values are shown in the bottom left of each panel.

Figure S8. Effect of sequence context on guide efficacy. Relative gRNA effect, the gRNA effect of a guide relative to the maximum gRNA effect in that guide set, is plotted on the y-axis. Average (solid line, y-axis) and median (individual dots) relative gRNA effect for guides targeting sequences with A (blue), C (green), G (red), or T (cyan) at bases 20bp upstream of the PAM site to 20bp downstream of the end of the targeting region (x-axis).

Figure S9. The effects of guide RNA secondary structure and DNA/RNA duplex formation strength (x-axis) on guide efficacy (y-axis). Each control guide RNA is one dot, large dots with connecting solid line denote median effect in sliding windows overlappping by 50%. A) TM calculated by Oligo TM B) ΔG of gRNA/DNA sequence's duplex predicted by RNA fold. C) TM of seed sequence (8 bases of guide nearest to PAM). D) ΔG of seed sequence gRNA/DNA duplex predicted by RNA fold . E) ΔG of the predicted secondary structure for the gRNA (including leader sequence "gtccctatcagtgatagagatggcgcacatggtacgctgtggtgctcgcggctgggaacgaaactctgggagctgcgattggcag" and the structural part of the gRNA).

Figure S10. As in Figure 5A but excluding 41 gRNAs directed against genes with inferred TSS positions. gRNA effect magnitude (absolute value of gRNA effects that were censored to have a maximum of 0) is plotted on the y-axis, against target position relative to the TSS on the x-axis. The median in 50bp windows (solid line, big markers), overlapping by 25bp, indicates a region of 200bp immediately upstream the TSS as effective.

Figure S1

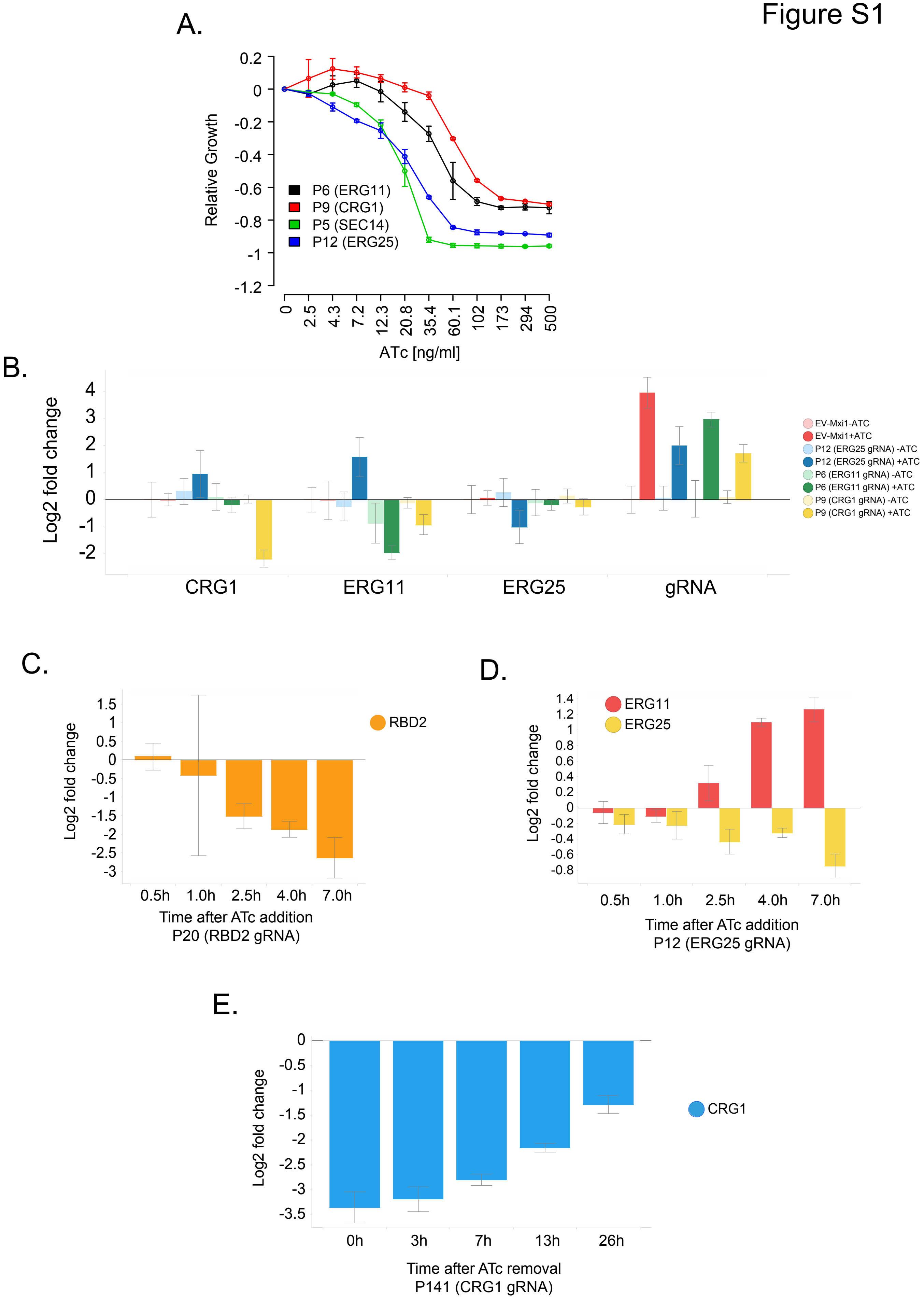
A.
Relative Growth
P6 (ERG11)
P9 (CRG1)
P5 (SEC14)
P12 (ERG25)
ATc [ng/ml]
0
2.5
4.3
7.2
12.3
20.8
35.4
60.1
102
173
294
500
B.
Log2 fold change
CRG1
ERG11
ERG25
gRNA
EV-Mxi1-ATC
EV-Mxi1+ATC
P12 (ERG25 gRNA) -ATC
P12 (ERG25 gRNA) +ATC
P6 (ERG11 gRNA) -ATC
P6 (ERG11 gRNA) +ATC
P9 (CRG1 gRNA) -ATC
P9 (CRG1 gRNA) +ATC
C.
Log2 fold change
RBD2
0.5h
1.0h
2.5h
4.0h
7.0h
Time after ATc addition
P20 (RBD2 gRNA)
D.
Log2 fold change
ERG11
ERG25
0.5h
1.0h
2.5h
4.0h
7.0h
Time after ATc addition
P12 (ERG25 gRNA)
E.
Log2 fold change
CRG1
0h
3h
7h
13h
26h
Time after ATc removal
P141 (CRG1 gRNA)

Figure S2

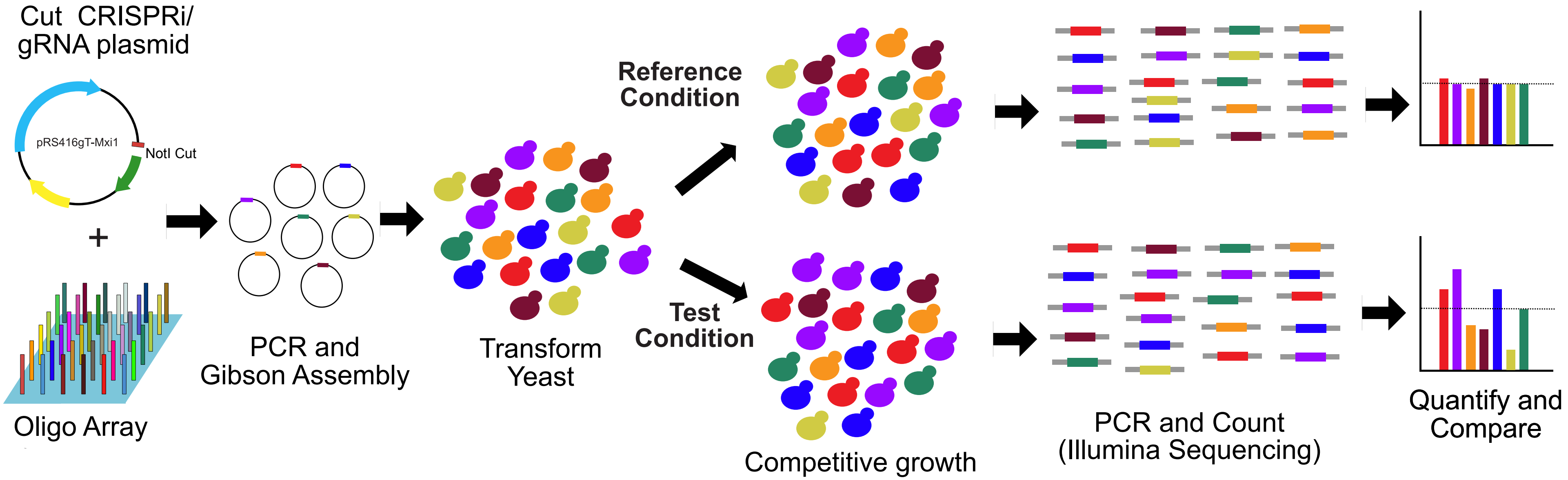

Conditions Compared		Metric Name	Abbreviation	Guides that are identified	Figures using this metric
**Test Condition**	**Reference Condition**				
**YPD +ATc**	**YPD –ATc**	ATc effect or guide effect*	*A0*	Highly-effective guides that repress a gene that is essential in YPD.	Figure 2A; 2B
**YPD +Drug +ATc**	**YPD +Drug –ATc**	ATc effect or guide effect*	*Adrug or A*	Highly-effective guides that repress a gene that is essential in YPD and additionally, less effective guides that moderately repress a gene that is dosage sensitive to the drug.	Figures 2B; 4; 5; S4A; S6; S7; S8; S9
**YPD +Drug +ATc**	**YPD –Drug +ATc**	Drug specific effect or drug effect	*D*	Only guides that repress a gene that is dosage sensitive to the drug.	Figure 2C,D,E; S4B,C;

*Guide effect, or gRNA effect, is the ATc effect (*A*) of a gRNA, calculated following growth in the presence of its specific partner reference compound.

Figure S3A

DMSO Replicates

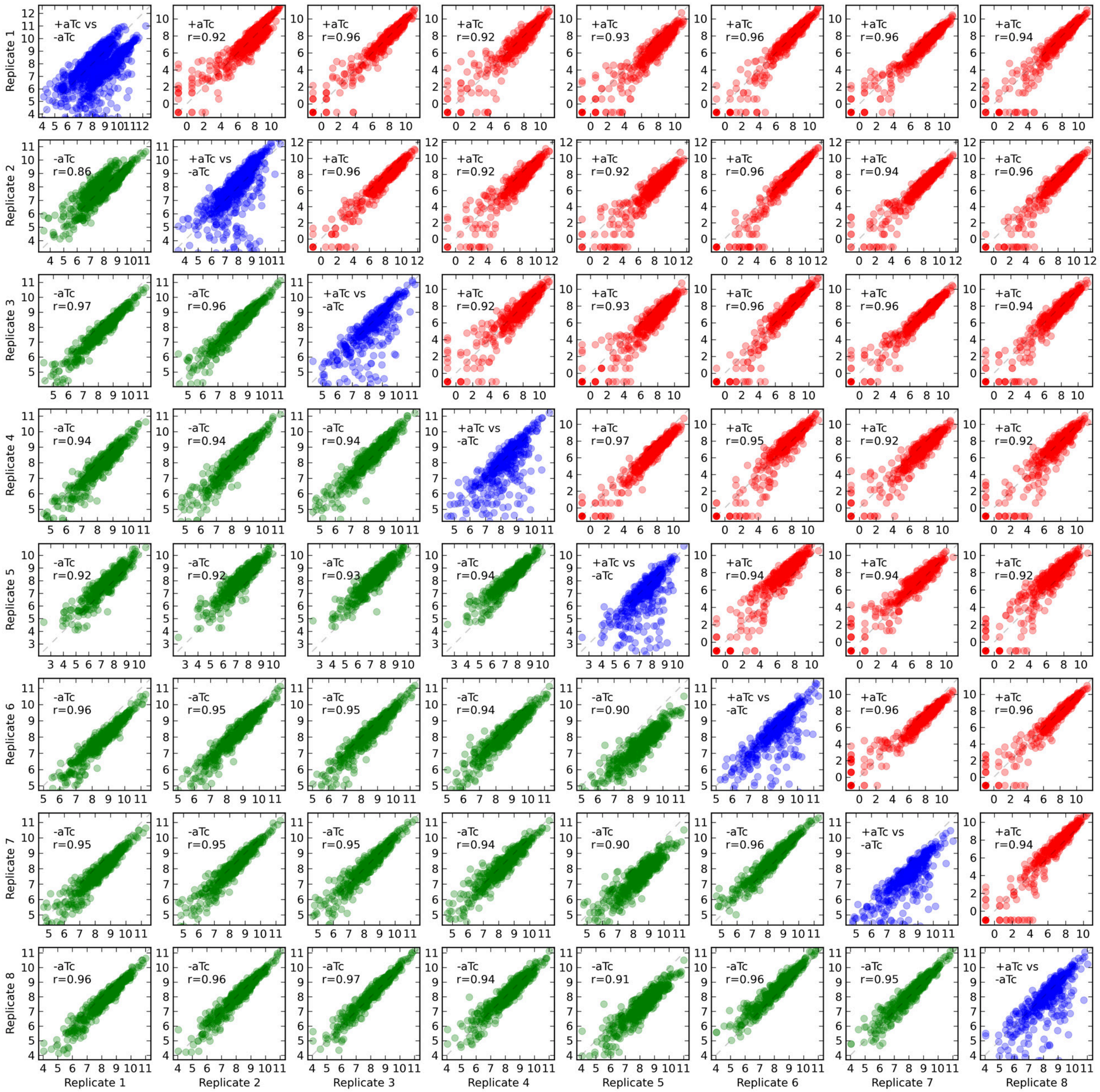

Figure S3B

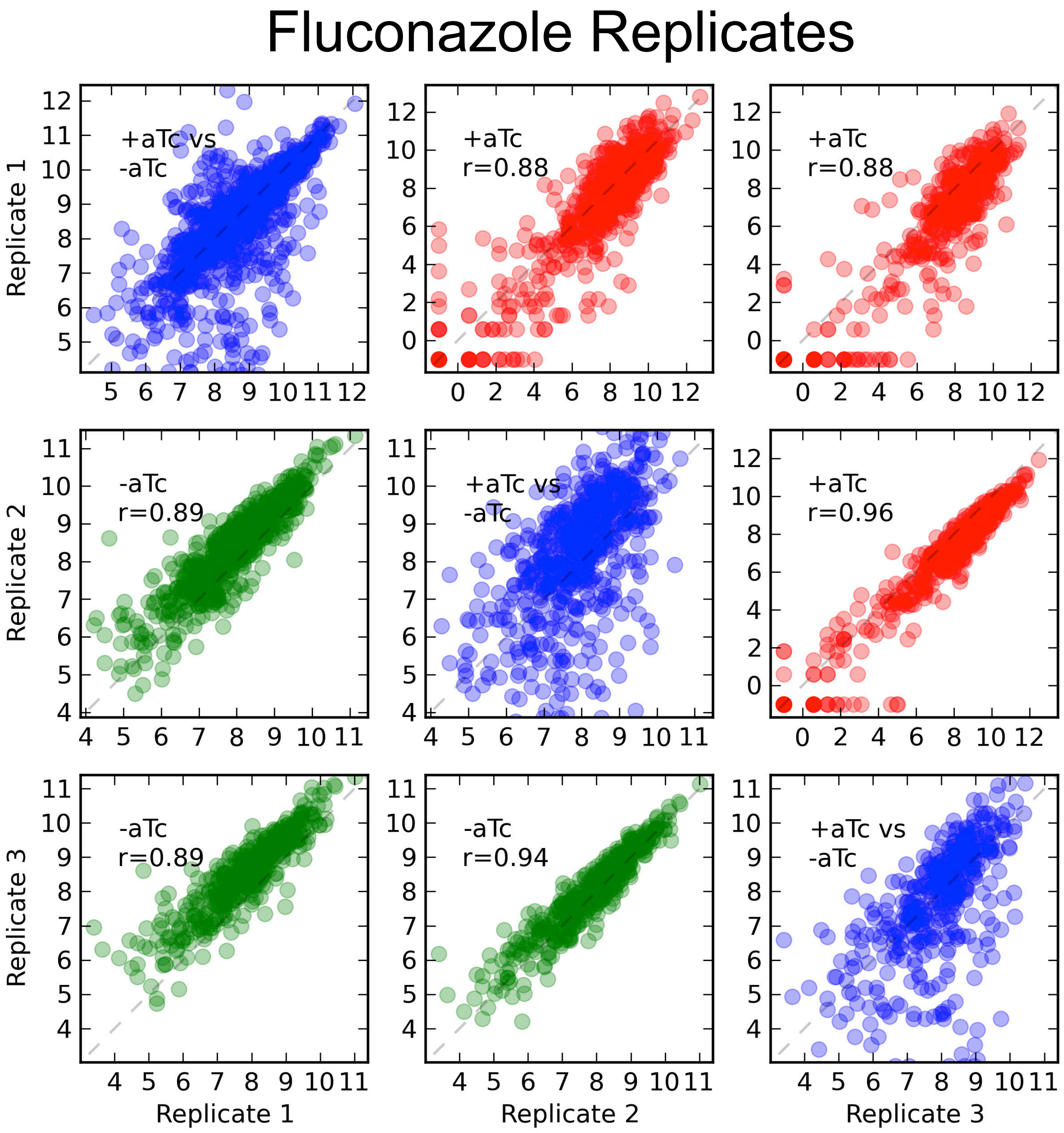
Fluconazole Replicates
+aTc vs
-aTc
+aTc
r=0.88
+aTc
r=0.88
-aTc
r=0.89
+aTc vs
-aTc
+aTc
r=0.96
-aTc
r=0.89
-aTc
r=0.94
+aTc vs
-aTc
Replicate 1
Replicate 2
Replicate 3
Replicate 1
Replicate 2
Replicate 3

Figure S4A

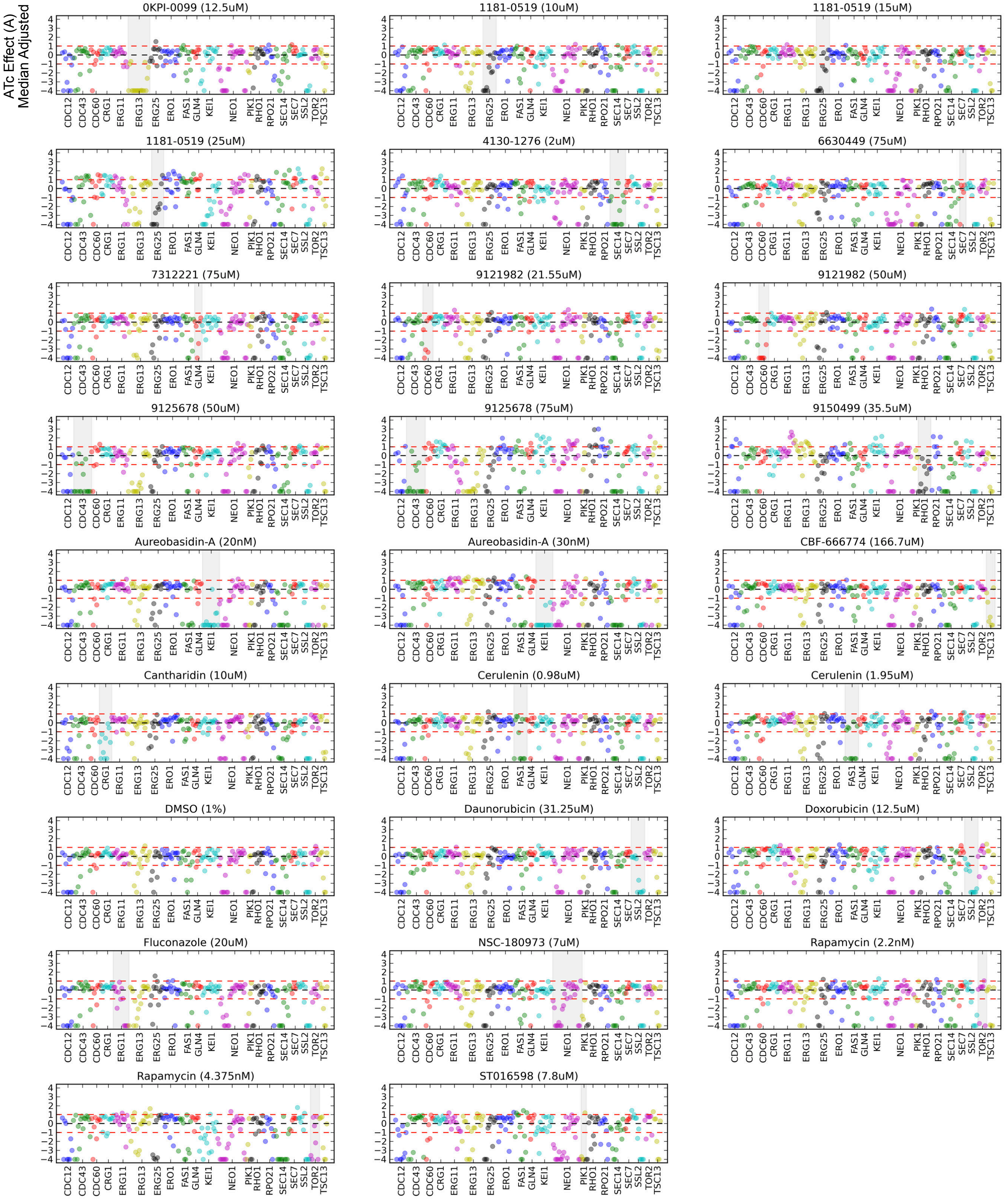

ATc Effect (A)
Median Adjusted
0KPI-0099 (12.5uM)
1181-0519 (10uM)
1181-0519 (15uM)
1181-0519 (25uM)
4130-1276 (2uM)
6630449 (75uM)
7312221 (75uM)
9121982 (21.55uM)
9121982 (50uM)
9125678 (50uM)
9125678 (75uM)
9150499 (35.5uM)
Aureobasidin-A (20nM)
Aureobasidin-A (30nM)
CBF-666774 (166.7uM)
Cantharidin (10uM)
Cerulenin (0.98uM)
Cerulenin (1.95uM)
DMSO (1%)
Daunorubicin (31.25uM)
Doxorubicin (12.5uM)
Fluconazole (20uM)
NSC-180973 (7uM)
Rapamycin (2.2nM)
Rapamycin (4.375nM)
ST016598 (7.8uM)
CDC12
CDC43
CDC60
CRG1
ERG11
ERG13
ERG25
ERO1
FAS1
GLN4
KEI1
NEO1
PIK1
RHO1
RPO21
SEC14
SEC7
SSL2
TOR2
TSC13

Figure S4B

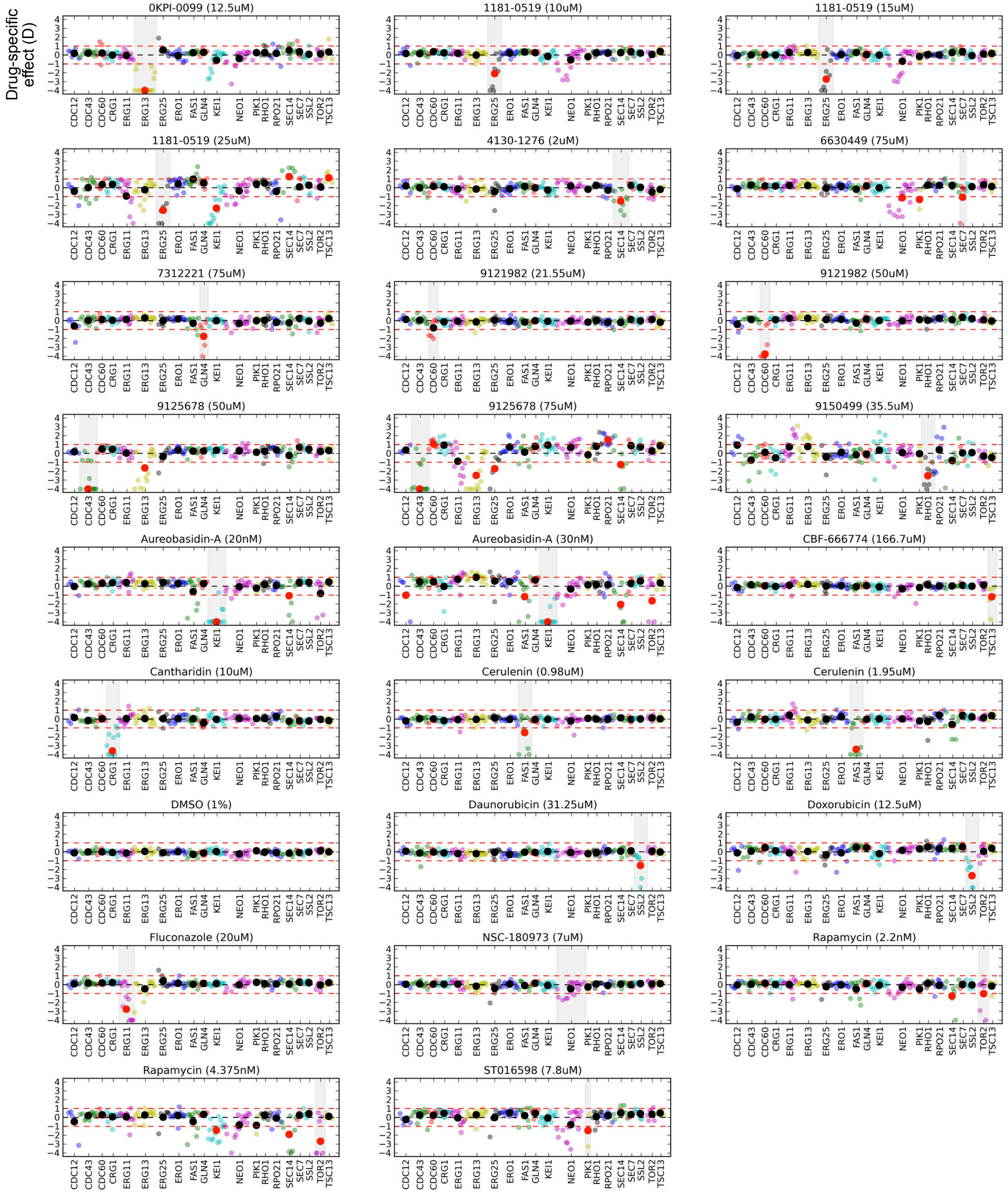

Drug-specific effect (D)
0KPI-0099 (12.5uM)
1181-0519 (10uM)
1181-0519 (15uM)
1181-0519 (25uM)
4130-1276 (2uM)
6630449 (75uM)
7312221 (75uM)
9121982 (21.55uM)
9121982 (50uM)
9125678 (50uM)
9125678 (75uM)
9150499 (35.5uM)
Aureobasidin-A (20nM)
Aureobasidin-A (30nM)
CBF-666774 (166.7uM)
Cantharidin (10uM)
Cerulenin (0.98uM)
Cerulenin (1.95uM)
DMSO (1%)
Daunorubicin (31.25uM)
Doxorubicin (12.5uM)
Fluconazole (20uM)
NSC-180973 (7uM)
Rapamycin (2.2nM)
Rapamycin (4.375nM)
ST016598 (7.8uM)
CDC12
CDC43
CDC60
CRG1
ERG11
ERG13
ERG25
ERO1
FAS1
GLN4
KEI1
NEO1
PIK1
RHO1
RPO21
SEC14
SEC7
SSL2
TOR2
TSC13

Figure S4C

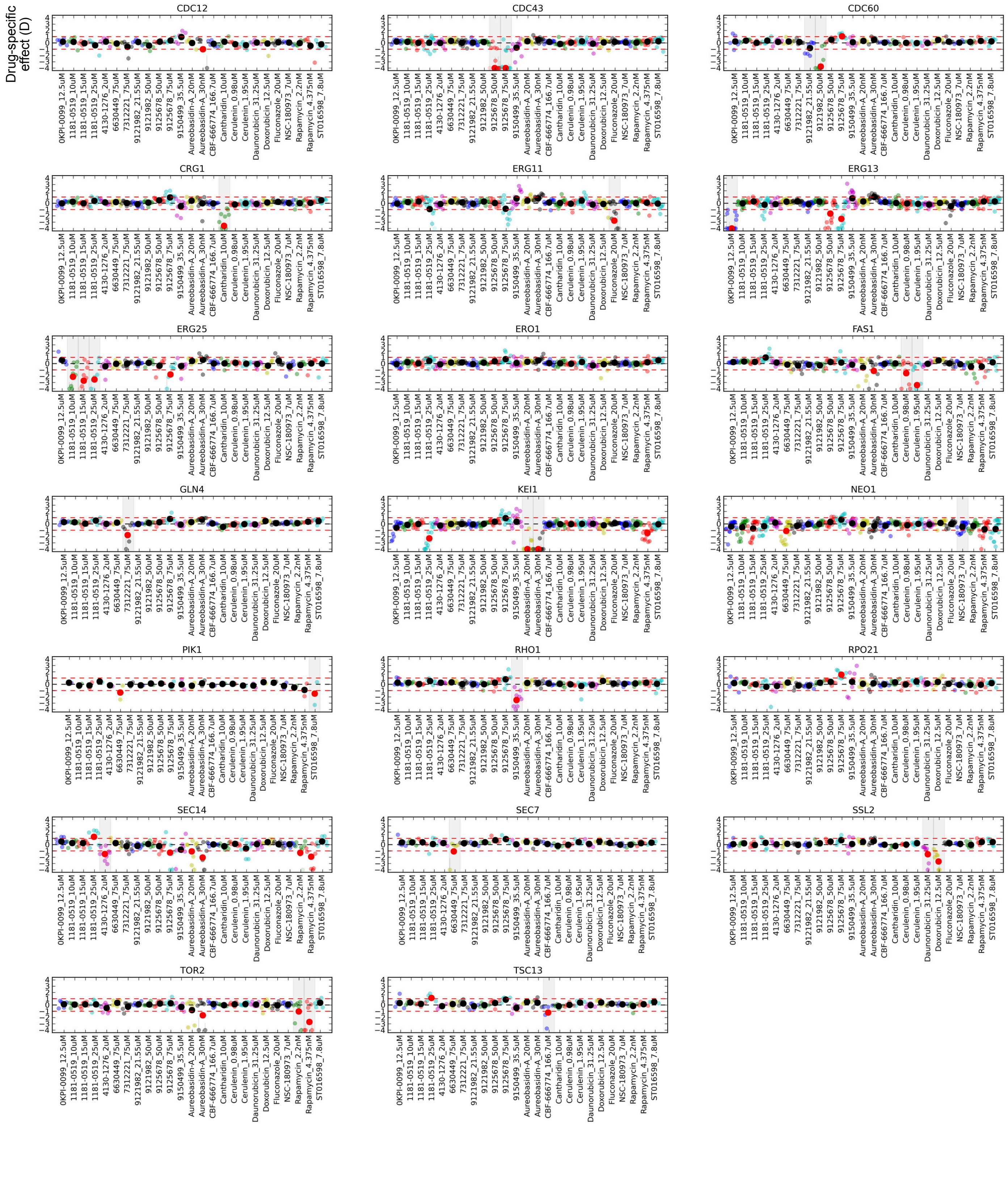
Drug-specific effect (D)
4
3
2
1
0
-1
-2
-3
-4
CDC12
CDC43
CDC60
CRG1
ERG11
ERG13
ERG25
ERO1
FAS1
GLN4
KEI1
NEO1
PIK1
RHO1
RPO21
SEC14
SEC7
SSL2
TOR2
TSC13
0KPI-0099_12.5uM
1181-0519_10uM
1181-0519_15uM
1181-0519_25uM
4130-1276_2uM
6630449_75uM
7312221_75uM
9121982_21.55uM
9121982_50uM
9125678_50uM
9125678_75uM
9150499_35.5uM
Aureobasidin-A_20nM
Aureobasidin-A_30nM
CBF-666774_166.7uM
Cantharidin_10uM
Cerulenin_0.98uM
Cerulenin_1.95uM
Daunorubicin_31.25uM
Doxorubicin_12.5uM
Fluconazole_20uM
NSC-180973_7uM
Rapamycin_2.2nM
Rapamycin_4.375nM
ST016598_7.8uM

Figure S5

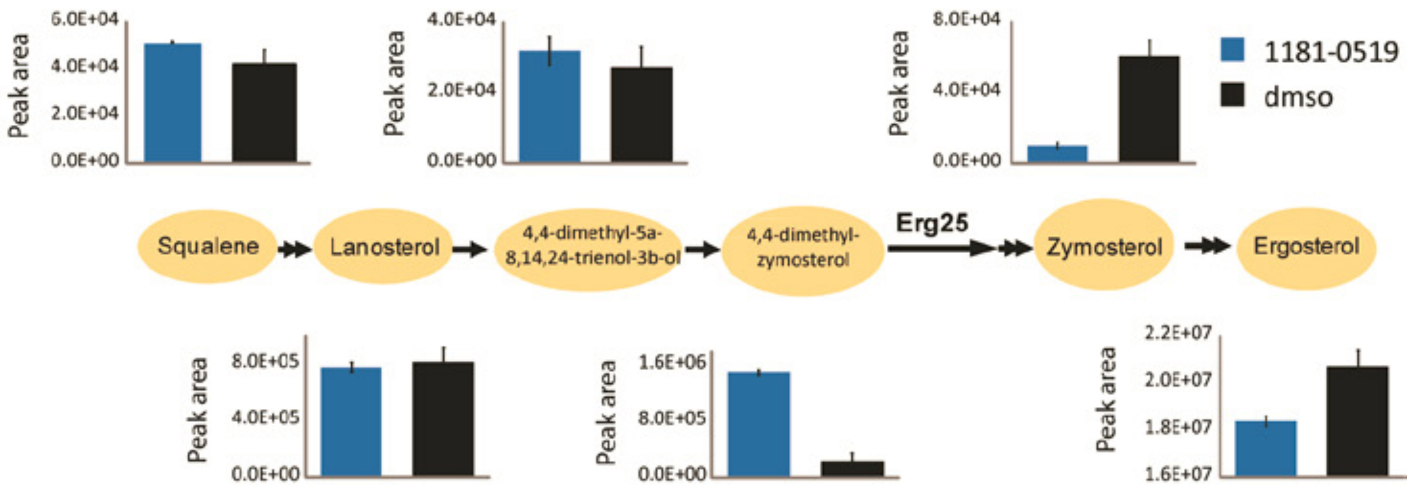

1181-0519
dmso
Peak area
6.0E+04
4.0E+04
2.0E+04
0.0E+00
Peak area
4.0E+04
2.0E+04
0.0E+00
Peak area
8.0E+04
4.0E+04
0.0E+00
Squalene
Lanosterol
4,4-dimethyl-5a-8,14,24-trienol-3b-ol
4,4-dimethyl-zymosterol
Erg25
Zymosterol
Ergosterol
Peak area
8.0E+05
4.0E+05
0.0E+00
Peak area
1.6E+06
8.0E+05
0.0E+00
Peak area
2.2E+07
2.0E+07
1.8E+07
1.6E+07

Figure S6

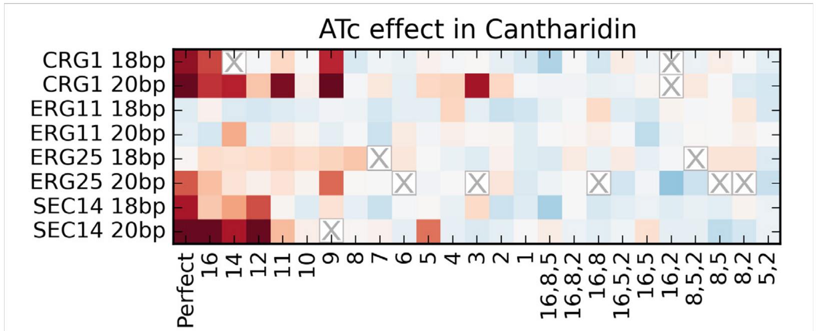

ATc effect in Cantharidin
CRG1 18bp
CRG1 20bp
ERG11 18bp
ERG11 20bp
ERG25 18bp
ERG25 20bp
SEC14 18bp
SEC14 20bp
Perfect
16
14
12
11
10
9
8
7
6
5
4
3
2
1
16,8,5
16,8,2
16,8
16,5,2
16,5
16,2
8,5,2
8,5
8,2
5,2

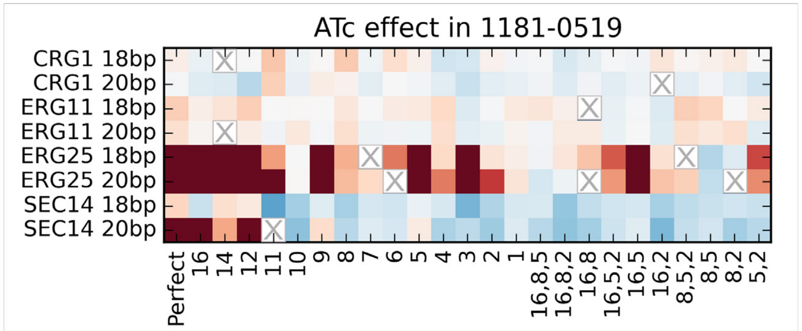

ATc effect in 1181-0519
CRG1 18bp
CRG1 20bp
ERG11 18bp
ERG11 20bp
ERG25 18bp
ERG25 20bp
SEC14 18bp
SEC14 20bp
Perfect
16
14
12
11
10
9
8
7
6
5
4
3
2
1
16,8,5
16,8,2
16,8
16,5,2
16,5
16,2
8,5,2
8,5
8,2
5,2

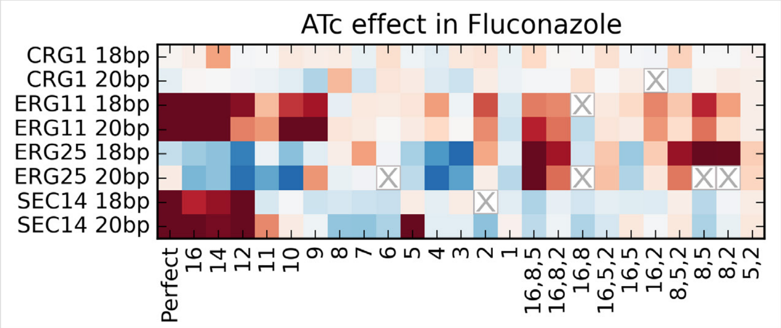

ATc effect in Fluconazole
CRG1 18bp
CRG1 20bp
ERG11 18bp
ERG11 20bp
ERG25 18bp
ERG25 20bp
SEC14 18bp
SEC14 20bp
Perfect
16
14
12
11
10
9
8
7
6
5
4
3
2
1
16,8,5
16,8,2
16,8
16,5,2
16,5
16,2
8,5,2
8,5
8,2
5,2

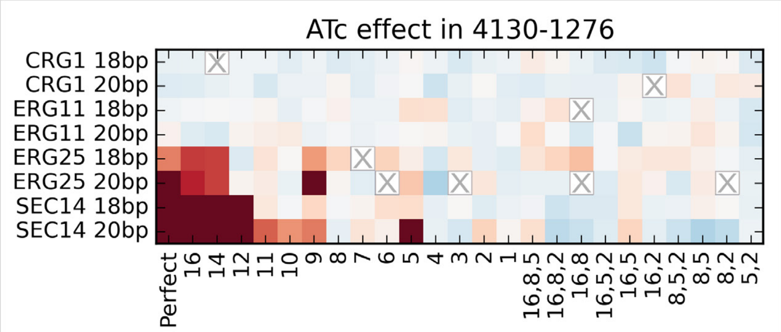

ATc effect in 4130-1276
CRG1 18bp
CRG1 20bp
ERG11 18bp
ERG11 20bp
ERG25 18bp
ERG25 20bp
SEC14 18bp
SEC14 20bp
Perfect
16
14
12
11
10
9
8
7
6
5
4
3
2
1
16,8,5
16,8,2
16,8
16,5,2
16,5
16,2
8,5,2
8,5
8,2
5,2

Figure S7

A.

B

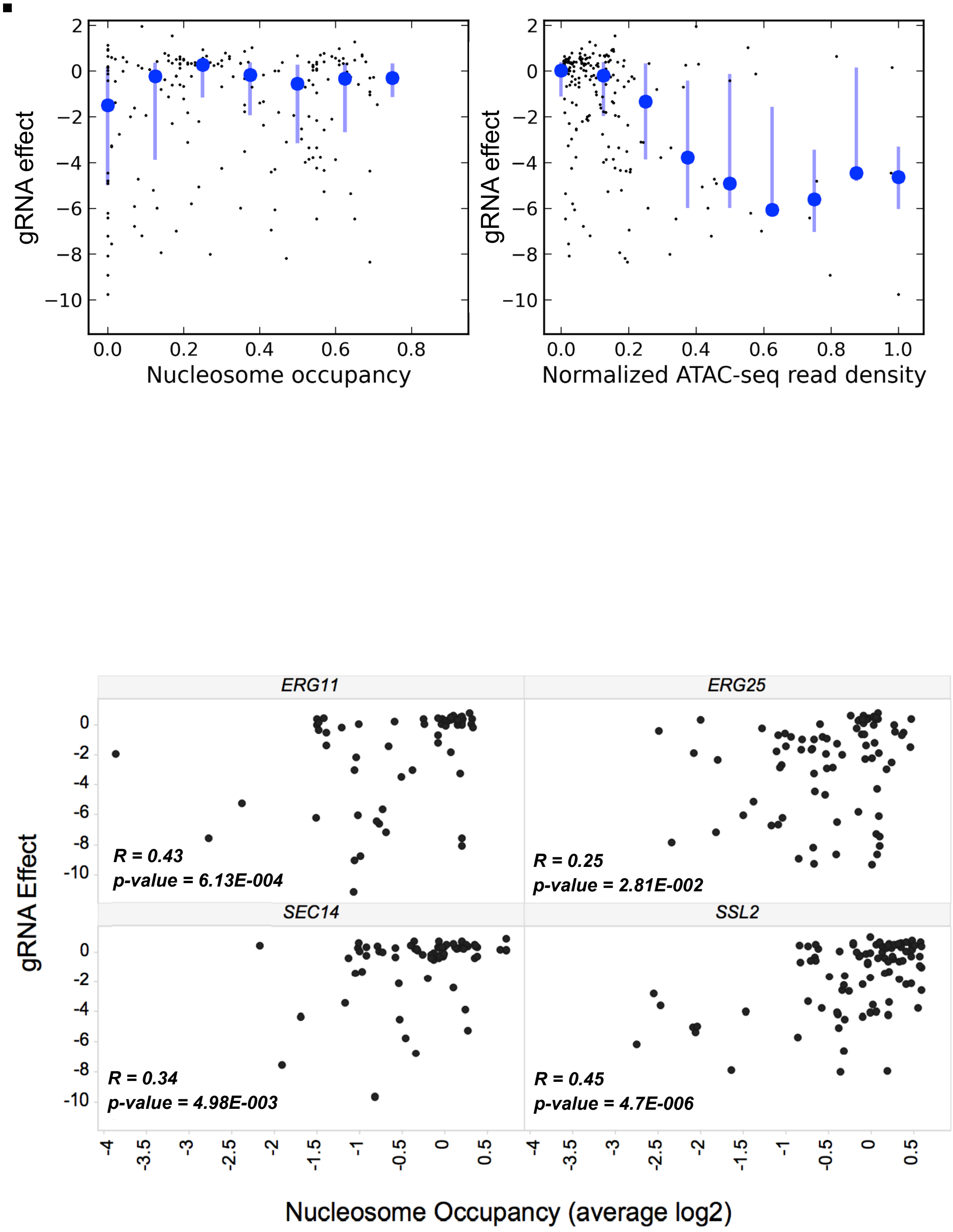
2
0
−2
−4
−6
−8
−10
gRNA effect
0.0
0.2
0.4
0.6
0.8
Nucleosome occupancy
1.0
Normalized ATAC-seq read density
ERG11
ERG25
SEC14
SSL2
R = 0.43
p-value = 6.13E-004
R = 0.25
p-value = 2.81E-002
R = 0.34
p-value = 4.98E-003
R = 0.45
p-value = 4.7E-006
gRNA Effect
-4
-3.5
-3
-2.5
-2
-1.5
-1
-0.5
0.5
Nucleosome Occupancy (average log2)

Figure S8

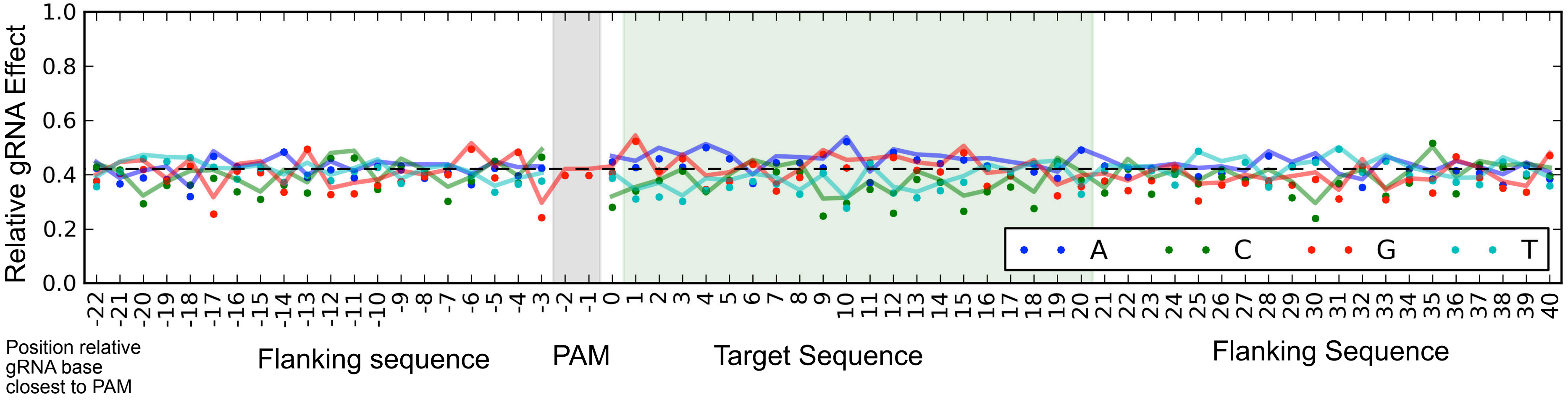
Relative gRNA Effect
1.0
0.8
0.6
0.4
0.2
0.0
A
C
G
T
-22 -21 -20 -19 -18 -17 -16 -15 -14 -13 -12 -11 -10 -9 -8 -7 -6 -5 -4 -3 -2 -1 0 1 2 3 4 5 6 7 8 9 10 11 12 13 14 15 16 17 18 19 20 21 22 23 24 25 26 27 28 29 30 31 32 33 34 35 36 37 38 39 40
Position relative gRNA base closest to PAM
Flanking sequence
PAM
Target Sequence
Flanking Sequence

Figure S9

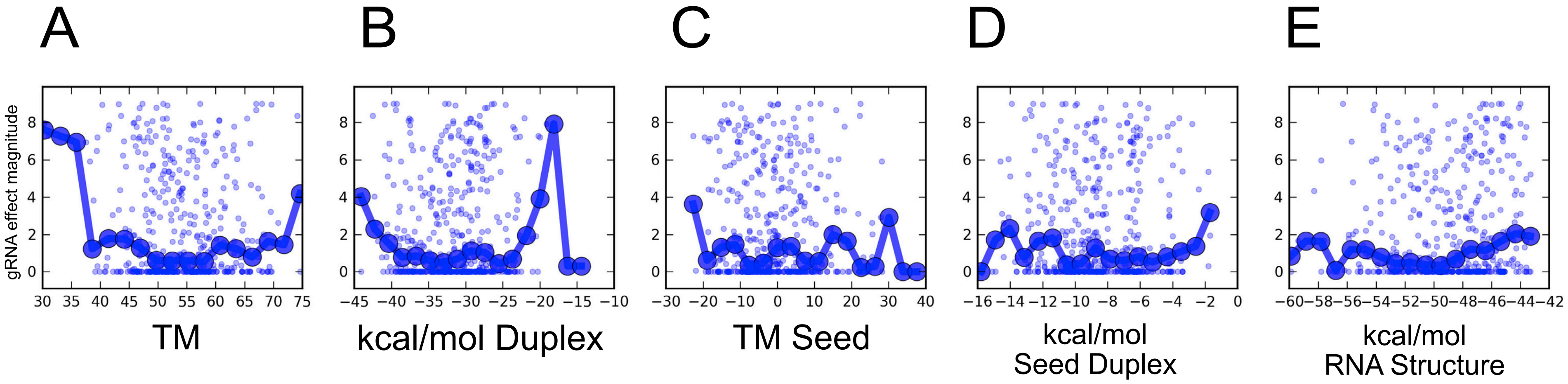

A
B
C
D
E
gRNA effect magnitude
0
2
4
6
8
30 35 40 45 50 55 60 65 70 75
TM
−45 −40 −35 −30 −25 −20 −15 −10
kcal/mol Duplex
−30 −20 −10 0 10 20 30 40
TM Seed
−16 −14 −12 −10 −8 −6 −4 −2 0
kcal/mol
Seed Duplex
−60 −58 −56 −54 −52 −50 −48 −46 −44 −42
kcal/mol
RNA Structure

Figure S10

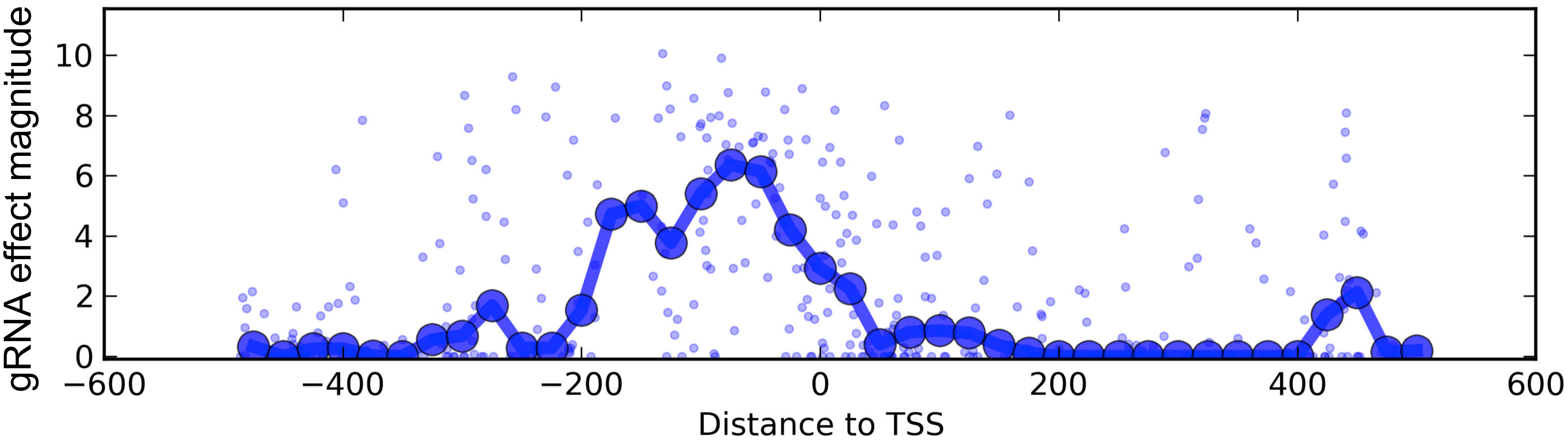

gRNA effect magnitude
10
8
6
4
2
0
−600
−400
−200
0
200
400
600
Distance to TSS
